# Lymphoma in Border Collies: Genome-Wide Association and Pedigree Analysis

**DOI:** 10.3390/vetsci10090581

**Published:** 2023-09-19

**Authors:** Pamela Xing Yi Soh, Mehar Singh Khatkar, Peter Williamson

**Affiliations:** 1 School of Life and Environmental Sciences, Faculty of Science, The University of Sydney, Camperdown, NSW 2006, Australia; pamela.soh@sydney.edu.au; 2 School of Medical Sciences, Faculty of Medicine and Health, The University of Sydney, Camperdown, NSW 2006, Australia; 3 Sydney School of Veterinary Science, Faculty of Science, The University of Sydney, Camperdown, NSW 2006, Australia; mehar.khatkar@sydney.edu.au; 4 School of Animal and Veterinary Sciences, The University of Adelaide, Roseworthy, SA 5371, Australia

**Keywords:** canine, cancer, lymphoma, GWAS, breed, Border Collie, dogs

## Abstract

**Simple Summary:**

Lymphoma is a common cancer in dogs, with large variation between breeds in both the incidence and frequency of immunophenotypes (B-cell or T-cell lymphoma). Very few studies have explored the genetic underpinnings of lymphoma in dogs, and it is not known whether various breeds share common risk genes considering the disparities in disease occurrence and immunophenotype distribution. In this study, our aim was to investigate lymphoma in a population of Border Collies, a breed at increased risk of lymphoma (often B-cell type). To our knowledge, this is the first genetic investigation of lymphoma risk in Border Collies. We examined pedigree data for possible inheritance patterns, and conducted a genetic investigation that incorporated the pedigree information to uncover possible genetic predispositions for lymphoma. We identified regions on chromosomes 18 and 27 that harbour cancer-related genes as prime candidates for lymphoma susceptibility, warranting further investigation.

**Abstract:**

There has been considerable interest in studying cancer in dogs and its potential as a model system for humans. One area of research has been the search for genetic risk variants in canine lymphoma, which is amongst the most common canine cancers. Previous studies have focused on a limited number of breeds, but none have included Border Collies. The aims of this study were to identify relationships between Border Collie lymphoma cases through an extensive pedigree investigation and to utilise relationship information to conduct genome-wide association study (GWAS) analyses to identify risk regions associated with lymphoma. The expanded pedigree analysis included 83,000 Border Collies, with 71 identified lymphoma cases. The analysis identified affected close relatives, and a common ancestor was identified for 54 cases. For the genomic study, a GWAS was designed to incorporate lymphoma cases, putative “carriers”, and controls. A case-control GWAS was also conducted as a comparison. Both analyses showed significant SNPs in regions on chromosomes 18 and 27. Putative top candidate genes from these regions included *DLA-79*, *WNT10B*, *LMBR1L*, *KMT2D*, and *CCNT1*.

## 1. Introduction 

Lymphoma is the most common haematological malignancy in dogs, with an incidence of about 13 to 114 per 100,000 dogs annually, and has varying incidence between countries and breeds [1,2,3,4]. The distribution of immunophenotypes is also highly varied across breeds, such that the T-cell type occurs in the majority of cases in some breeds (e.g., Siberian Husky) while the B-cell type occurs in the majority of cases in others (e.g., Basset Hound and Rottweiler) [5,6]. 

Canine lymphoma has numerous parallels to human non-Hodgkin’s lymphoma, potentially implicating the shared environment on the aetiology of the disease [7,8]. The aetiology of canine lymphoma is not fully understood and is likely a result of complex interactions between genetics and the environment. Previous studies have explored chromosomal aberrations [9], epigenetic silencing [10] epigenetic deregulation [11], long non-coding RNAs [12], microRNAs [13], DNA methylation [14], somatic mutations [15], and germline mutations [16] as mechanisms for canine lymphoma. 

Several environmental risk factors have been identified, such as exposure to herbicides [17], tobacco smoke [18], or household chemicals [19] or proximity to industrial areas [20] and waste management [6,21]. Geographic associations with the incidence of canine lymphoma have also been explored and show similarities to human lymphoma incidence [4,17].

Several studies have exploited genome-wide association studies (GWASs) to identify risk genotypes for canine lymphoma. One study reported two shared risk loci on chromosome 5 for B-cell lymphoma and hemangiosarcoma but did not find any single nucleotide polymorphisms (SNPs) significantly associated with B-cell lymphoma alone [22]. Another study detected a significant signal on chromosome 4 and identified the candidate genes *MCC*, *MXD3,* and *FGFR4* [23]. The authors were unable to find any SNPs significantly associated with lymphoma when a GWAS was conducted across multiple breeds [23]. A study of T-zone lymphoma found genome-wide significance on chromosome 8, near thyroid hormone regulation genes *DIO2* and *TSHR*, and on chromosome 14, near hyaluronidase genes *SPAM1*, *HYAL4*, and *HYALP1* [24]. Given the phenotypic and genetic diversity across dog breeds [25,26,27,28,29,30,31,32,33,34] and differences in the incidence and immunophenotype of lymphoma [1,2,3,4,5,6], it is not yet clear whether genetic risk factors for lymphoma in one breed will be the same in another. A more recent study of multiple breeds with a range of haematopoietic malignancies showed some overlap in associated regions and the potential for pleiotropic effects [35].

Border Collies are a popular breed worldwide and are at increased risk of lymphoma compared to the general population [36]. Lymphomas in Border Collies are most often of the B-cell immunophenotype (84 to 91% of cases) [5,37]. We previously reported the results of a survey of lymphoma of Border Collies in Australia and identified 28 cases with a common female ancestor [37], suggesting a potential heritable risk. In this study, the case number and extent of pedigree analysis was expanded and used to inform SNP-based GWAS analyses. 

## 2. Methods 

### 2.1. Animals 

This study was conducted in accordance with guidelines from the Animal Research Act, NSW, Australia, approved by the Animal Ethics Committee of the University of Sydney under ethics numbers 37/634/6013. A total of 289 blood samples (38 cases, 251 controls) from Border Collies were collected in EDTA-coated vacutainers by licensed veterinarians and were voluntarily submitted from owners with written informed consent. Dogs were considered ‘purebred’ if they had been registered by the Australian National Kennel Club (ANKC) or following visual inspection by veterinarians. Diagnosis of lymphoma was confirmed through immunohistochemistry, with T- or B-cell subtype reported for 22 of the 38 lymphoma cases. Controls were from samples in an in-house biobank that were reported to be lymphoma-free at the time of collection, and 11 were confirmed by owners to not have lymphoma at the time of a health survey [37]. Further details of dogs are included in Supplementary Data S1.

### 2.2. Pedigree Construction 

Pedigree data were available through an extensive database with records from over 83,000 Border Collies [30]. Clinical details of 71 lymphoma cases and pedigree information for these dogs were provided with consent, either through survey [37] or via direct reporting from owners. Pedigrees were constructed using a combination of the R packages ‘kinship2’, ‘pedigree’, and ‘FamAgg’ [38,39,40]. 

### 2.3. Genotyping and Imputation 

DNeasy Blood and Tissue Kit (Qiagen, Melbourne, Victoria, Australia) was used to isolate genomicDNA following the manufacturer’s protocol. Genotyping was conducted by Geneseek (Lincoln, NE, USA) using the CanineHD BeadChip (Illumina, San Diego, CA, USA). Sixty-one DNA samples were genotyped on an older array covering over 170,000 evenly spaced, genome-wide SNPs (170 k dataset), while 230 samples were genotyped on a more extensive array covering over 220,000 SNPs (220 k dataset). Genotype data were imputed, using the same methods as those described in [30] to match all samples to the higher-density SNP chip using BEAGLE v5.1 [41]. Both datasets were first filtered separately using PLINK v1.9 [42] for individual missingness of 0.2 (--mind 0.2), and the 220 k dataset was filtered for a minor allele frequency of 0.02 (--maf 0.02). The datasets were then merged and filtered to remove SNPs with over 25% missing call rate (--geno 0.25), then imputed on BEAGLE v5.1 [41] with default settings except that the cluster was set to 0.05 and effective population was set to 100. An effective population size of 100 was selected as pedigree estimates of diversity suggest an effective population size of 99 to 129 for Border Collies [30,32,43].

### 2.4. Genome-Wide Association Study (GWAS) 

Two cases were excluded from the GWAS due to uncertainties with their diagnosis; whether these dogs had stage V lymphoma (includes any stage I-IV signs and bone marrow involvement; secondary leukaemia) or acute lymphocytic leukaemia (ALL) (neoplastic lymphocytes or lymphoid progenitors originating in the bone marrow) was never elucidated. Stage V lymphoma and ALL can be difficult to differentiate and the prognosis and response to treatment for each disease is different [44,45], which may suggest different molecular causes. Since 84 to 90% of lymphoma cases in Border Collies are B-cell type [5,37], and T-cell non-Hodgkin’s lymphoma (NHL) in humans has different genetic causes from B-cell NHL [46], another 4 dogs diagnosed with T-cell lymphomas were excluded. Of the remaining 32 lymphoma cases, 18 were B-cell lymphomas, while the rest were untyped. 

As there were many relatives of lymphoma cases in the control population and to improve power, a quantitative phenotype was chosen, based on the assumption of Mendelian inheritance for a recessive risk genotype for lymphoma, such that lymphoma cases were coded as 2, carriers were coded as 1, and controls were coded as 0. Carriers included dogs that were offspring of lymphoma cases, parents of lymphoma cases, or grandparents/great-grandparents of at least two cases. PIHAT values (proportion of identity-by-descent (IBD)) were calculated in PLINK v1.9 using --genome [42]. Controls with PIHAT > 0.25 (i.e., first- and second-degree relatives) to cases were also removed. There was a total of 27 carriers included in the analysis, including 10 offspring of lymphoma cases, 12 parents of one case each (6 of which were also grandparents or great-grandparents of one or more cases), 1 parent of two cases, and 4 dogs that were grandparents or great-grandparents of at least two cases. Out of the 221 controls that remained after genotype quality control filtering, 22 dogs were removed due to their relationship (known through pedigree) with lymphoma cases as it was unclear whether they could be carriers for a risk genotype. This included one great-grandparent, eight grandparents, five siblings, six half-siblings, one grandchild, and one aunt of lymphoma cases. A separate GWAS was also run using just the cases and controls (excluding those with PIHAT > 0.25 to cases) with a binary phenotype to compare these results to the results from the quantitative phenotype GWAS. 

Genome-wide Complex Trait Analysis (GCTA) [47] was used to filter the imputed dataset for a minor allele frequency of 0.02 and create genetic relationship matrices (GRM), which were incorporated into the mixed linear model analysis (MLMA). All plots were generated using R v3.6.1 (R Development Core Team 2011). False discovery rates (FDR) were estimated by genome-wide q-values and chromosome-wise q-values through the R package “qvalue” using the MLMA output of *p*-values [48]. A q-value threshold of 0.1 was selected based on sample size and power estimate. Manhattan plots were created in R using the R package “qqman” [49]. A linear discriminant analysis (LDA) for the top 100 SNPs (ranked by *p*-value) for each GWAS was used to find the combination of SNPs that best explained the phenotype and discriminated between cases and controls through a backwards stepwise regression. Carriers were removed from the quantitative phenotype GWAS dataset. This was conducted using a linear model for phenotype (1 for controls and 2 for cases) as the outcome variable and using the genotypes of cases and controls at the significant SNPs as the predictor variables. The output was then used in the inbuilt R package ‘stats’ function step(), with direction set to ‘backward’. 

Regional association plots were generated using modified code from R packages “RACER” and “IntAssocPlot” [50,51]. Linkage disequilibrium (LD) values (r^2^) for the regional association plots were calculated using PLINK v1.9 [42] and gene annotations were obtained from the National Center for Biotechnology Information (NCBI) (GCF_000002285.3 CanFam3.1, retrieved on 8 January 2020). Genes in all cancer pathways and viral infection pathways for *Canis familiaris* were fetched from KEGG (http://www.kegg.jp/, accessed on 8 January 2020) using the R package “KEGGREST” [52]. Viral infection pathway genes were included based on studies of lymphoma and viruses in humans [53,54,55]. Ensembl (https://www.ensembl.org, accessed on 8 January 2020) Release 106 dog genome CanFam3.1 (GCA_000002285.2) [56] was used to investigate genes of interest and genetic variants that are predicted to affect protein function using Sorting Intolerant From Tolerant (SIFT) values [57]. SIFT values less than 0.05 indicate that the variant in question likely affects protein function. Variants were also investigated in the dog population from [27], integrated in Ensembl as PRJEB24066. 

### 2.5. Haplotype Analysis 

Haplotype association analyses were conducted by creating haplotype blocks with PLINK v1.9 [42] for significant chromosomes in the GWAS and then using the haplotype blocks in PLINK v1.07 [58] as input for --hap. The quantitative phenotype was used for a linear regression (--hap-linear) while the binary phenotype was used for a logistic regression (--hap-logistic) for cases and controls. Haplotype frequencies for each group and each highly significant block (*p* < 0.0001) were calculated using a custom R script. 

### 2.6. Relationship Networks (NetView) 

The R packages ‘netview’ and ‘GGally’ were used to construct relationship networks based on pedigree or genotype data and clustered based on a *k*-value of 10 [59]. Interactive networks were created using the R package ‘network D3’ [60]. To visualise the relative position of each lymphoma case in the population and to investigate case clustering, usage of pedigree data was maximised by creating a kinship matrix based on 15 generations of ancestry for dogs with available health status from our previous survey, as well as all genotyped dogs that had pedigrees available. This included 54 lymphoma cases (including 22 genotyped), 114 normal controls (12 genotyped), and 40 other genotyped dogs of unknown health status. An additional relationship network was created based on genotype data using a distance matrix created in PLINK v1.9 [42] for only the dogs included in the GWAS analysis, including 31 lymphoma cases, 27 carriers, and 119 controls. 

### 2.7. Restricted Maximum Likelihood (REML) Analysis 

A REML analysis was conducted for the cases (31 dogs) and controls (119 dogs) in GCTA [47] to estimate the phenotypic variance (Vp) explained by the genetic variance (V(G)) for (1) all the autosomes together, (2) each autosome separately, and (3) significant regions from the GWAS output. The analysis uses a log likelihood ratio test to calculate *p*-values. The variance explained (V(G)/Vp, heritability) is transformed to the underlying liability scale (V(G)/Vp_L) using disease prevalence such that if heritability on the observed scale is very large, this value becomes relatively small when transformed to the liability scale if there is ascertainment bias (i.e., proportion of cases to controls is much larger than prevalence), which is typical in case-control studies [61]. Previous reports from South Africa estimated a lymphoma prevalence of 0.02 in Border Collies [62]. Since the prevalence of lymphoma in Border Collies in Australia has not been reported, prevalence values of 0.1, 0.05, and 0.025 were tested for each analysis. 

## 3. Results 

### 3.1. Pedigree Investigations 

**Figure 1:**
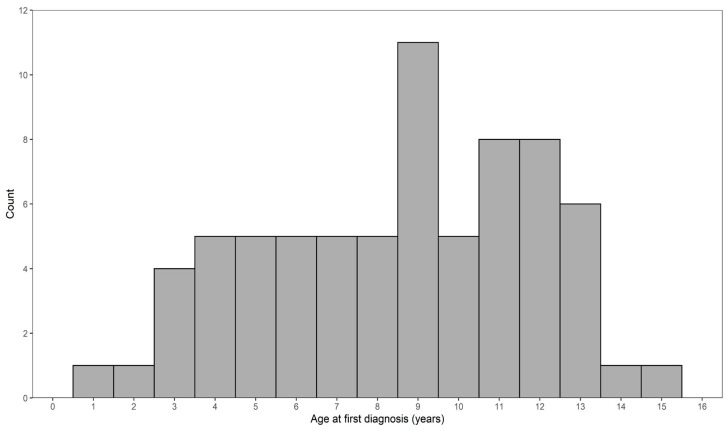
Distribution of age at first diagnosis for 71 lymphoma cases.

Along with the 35 genotyped lymphoma cases, an additional 30 lymphoma cases that had pedigree information were known from our previous health survey study [37], and another six deceased dogs that were relatives of genotyped or surveyed lymphoma cases that had also been diagnosed with lymphoma were reported privately to us from owners. Of these 71 dogs, 25 had B-cell lymphomas (35%), three had T-cell lymphomas (4%), and 43 had unknown type lymphomas (61%). The mean age at first diagnosis was 8.7 years (SD 3.3). The distribution of age at first diagnosis of lymphoma is presented in Figure 1. The peak age at diagnosis was 9 years, while the age at diagnosis ranged from as young as 1 year and 11 months to 15 years and 3 months. 

**Figure 2:**
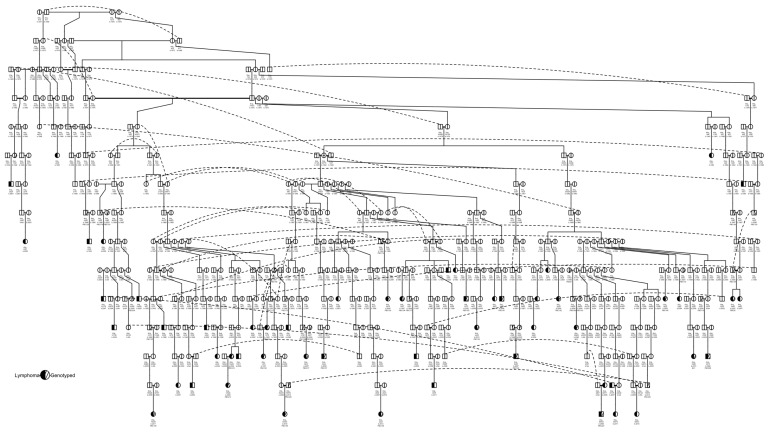
Pedigree of 54 lymphoma cases descending from dog_811. The most direct pathway for each lymphoma-affected dog to dog_811 is shown; some individuals may be related through other pedigree pathways but these pathways are not shown. Dotted lines connect instances where an individual is repeated in the pedigree. The year of birth and genotype ID, if available or relevant, is indicated below the dog ID for each dog.

Of these 71 cases, 54 had pedigrees available (22 genotyped). All 54 pedigree cases were traced to a common ancestor born in 1968, dog_811 (Figure 2). The common ancestor for 28 cases identified in our previous study [37], dog_67066, was an offspring of dog_811, and was identified as an ancestor to 52 of these cases. In this pedigree, there were two pairs of full-sibling littermates diagnosed with lymphoma (dog_13422 and dog_13511; dog_2445 and dog_2446). One of the affected littermates, dog_13422, was also a parent of another case, dog_13339, and was one of five affected parent–offspring duos. One of the other affected parent–offspring duos (dog_33176 and dog_30475) descended from dog_50125, which sired two other cases to separate dams and was a grandparent of two cases and a great-grandparent of one case. One parent (dog_2625) in another affected parent–offspring duo (dam dog_2625 and offspring dog_2626) had an affected aunt (dog_2615) that was a full sibling of dog_2625′s dam. The sire of dog_2615 and dog_2625′s dam was also a grandparent of another case (dog_2629). Dog_50681, which we previously identified as a common ancestor for 12 cases [37], was identified as a common ancestor for an additional seven cases. These 19 cases primarily descended from two full siblings, dog_44286 (four cases) and dog_44277 (12 cases), while three dogs descended from dog_50681. Within these 19 cases, there were three pairs of parent–offspring cases and one aunt–niece occurrence of lymphoma.

**Figure 3:**
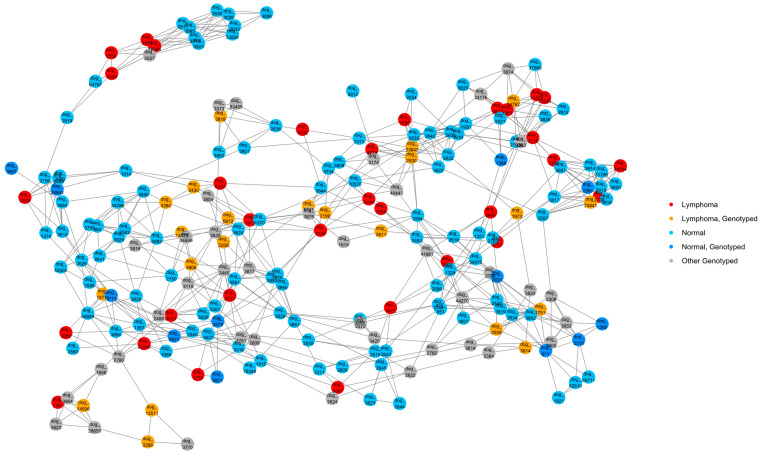
NetView relationship network of 215 dogs based on a pedigree kinship matrix. Clustered based on a *k*-value of 10. A total of 54 lymphoma cases (22 genotyped), 114 normal (12 genotyped) dogs, and 40 other genotyped dogs with unknown health status are shown.

A NetView plot was created using a pedigree kinship matrix with the 54 known lymphoma cases, normal pedigree dogs identified through our previous health survey, and genotyped dogs that had pedigrees available (Figure 3). The plot showed that lymphoma cases were widespread in the network, and a good distribution of animals was captured in the genotyping. There was one cluster of dogs that separated from the main network (top left of figure), which included four lymphoma cases, none of which were available for genotyping.

### 3.2. GWAS with Quantitative Phenotype 

Through pedigree mapping, we were able to identify the relationships of genotyped dogs with all known lymphoma cases and the familial occurrence of lymphoma that suggested a potential heritable risk. This information was used to design the GWAS through the assumption of Mendelian inheritance of a recessive genotypic risk for lymphoma. Putative “carriers” were identified as dogs that were either (i) parents of lymphoma cases; (ii) offspring of lymphoma cases; or (iii) grandparents or great-grandparents of at least two cases. Dogs that could potentially be “carriers”, such as siblings or half-siblings of lymphoma cases or grandparents and/or great-grandparents of only one case, were removed from the GWAS analysis due to the uncertainty of their status as “carrier” or control. After the imputation and filtering of the genotype data, as well as the exclusion of cases based on uncertain or T-cell diagnoses and the exclusion of controls due to relationships with lymphoma cases (PIHAT > 0.25), 177 dogs (31 lymphoma cases, 27 carriers, and 119 controls) and 153,900 SNPs were left for genomic analyses. 

**Figure 4:**
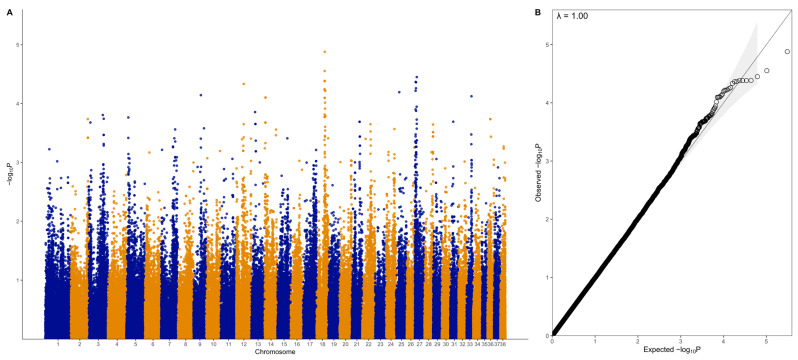
Genome-wide association analysis results for the quantitative phenotype (31 lymphoma cases, 27 carriers, and 119 controls). (**A**) Manhattan plot of −log_10_ *p*-values from mixed linear model association analysis. (**B**) QQ-plot of expected and observed −log_10_ *p*-values; shaded area indicates 95% confidence interval.

**Figure 5:**
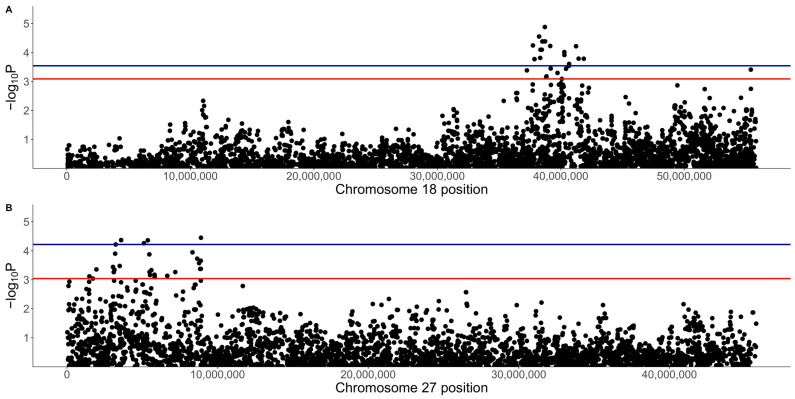
Regional Manhattan plots for the quantitative phenotype results on (**A**) chromosome 18 and (**B**) chromosome 27. The blue line indicates a chromosome q-value cut-off of 0.05 and the red line indicates a q-value cut-off of 0.1.

**Figure 6:**
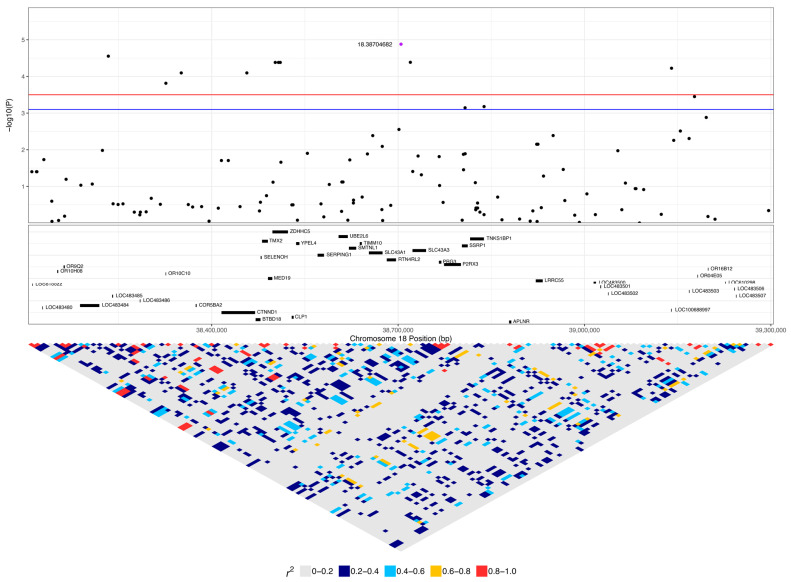
Regional association plot for the most significant region on chromosome 18. The top of the plot indicates −log10 *p*-values for each SNP from the mixed linear model association analysis. Red and blue lines indicate q-value cut-offs of 0.05 and 0.1, respectively. The top associated SNP for this chromosome, 18:38704682, is highlighted in purple. The protein-coding genes (CanFam3.1, assembly GCF_000002285.3) in the region, fetched from NCBI, are shown in the middle box. Linkage disequilibrium (r2) in the region is shown in the bottom triangle.

There were no genome-wide significant SNPs (q < 0.1) from the mixed linear model analysis (Figure 4); however, chromosome-wise calculated q-values showed significance (q < 0.1) for chromosomes 18 and 27 (Figure 5). Chromosome 18 was significant for 28 SNPs, with 27 of these SNPs being located between 37.2 Mb and 41.9 Mb and one SNP at 55.4 Mb (Figure 4A, Table 1). Three of these SNPs were within *ZDHHC5*, and there was one SNP each in *LOC100688997*, *LOC100684610*, *CTNND1*, *OR10C10*, *OR5B21*, *TMEM109*, *LOC483451*, and *SSRP1*. To account for SNPs in linkage, the significant region of interest was expanded 500 kb upstream and downstream to search for relevant genes. The 36.7 to 42.4 Mb region spanned a total of 238 protein-coding genes, including five genes that were listed in cancer or viral infection pathways from KEGG. These genes were *DLA-79*, *PSMC3*, *DDB2*, *NR1H3,* and *SPI1*. The genes *PSMC3*, *DDB2*, *NR1H3,* and *SPI1* were downstream of the significant region (42.2–42.4 Mb). The top associated SNP, 18:38704682, was in a non-coding region but had low to moderate linkage (0.2 < r^2^ < 0.5) with nine SNPs (Figure 6, Table 1). Eight lymphoma cases carried homozygous AA at this SNP. Of these eight, six had confirmed B-cell lymphoma diagnosis. Four cases were in complete remission (disappearance of tumours and symptoms) following a chemotherapy protocol, two were in partial remission and were still undergoing treatment, one survived a month after initial diagnosis and treatment with chemotherapy, and one had palliative care and was euthanised a month after initial diagnosis. The ages at first diagnosis for these eight dogs ranged from 3 years to 11 years and 5 months. The nine SNPs linked to this top SNP spanned the genes *SLC43A1*, *SLC43A3*, *SSRP1*, and *PRG3*. Two of these SNPs were in non-coding regions. Two other SNPs were in *SLC43A1* (18:38659203 and 18:38674512), one of which was within exon 15 and is a 3′ UTR variant (18:38674512). *SLC43A1* has two missense variants that are not tolerated (SIFT = 0), G > A at 18:38665640, causing G191S, and C > T at 18:38663900, causing P217S. Three SNPs (18:38732149, 18:38737451, 18:38723578) were within *SLC43A3*, one of which was within exon 1 and is a 5′ UTR variant (18:38723578). *SLC43A3* has one missense variant, a T > C mutation at 18:38728209, causing C56R (SIFT = 0.03). One SNP (18:38805092) was in exon 5 of *SSRP1* and is a splice region variant. There was another SNP in *SSRP1* (18:38807806) that was significant in the analysis (Table 1), and homozygosity (AA) at this SNP occurred in two cases and not in carriers or controls. *SSRP1* has three missense variants that are tolerated (SIFT > 0.05). The remaining linked SNP (18:38766110) was in exon 1 of *PRG3* and is annotated as a missense variant (C > T), causing L6F with a SIFT value of 0.07. *PRG3* has another missense variant at 18:38766791 but is also tolerated (SIFT = 0.09). 

**Figure 7:**
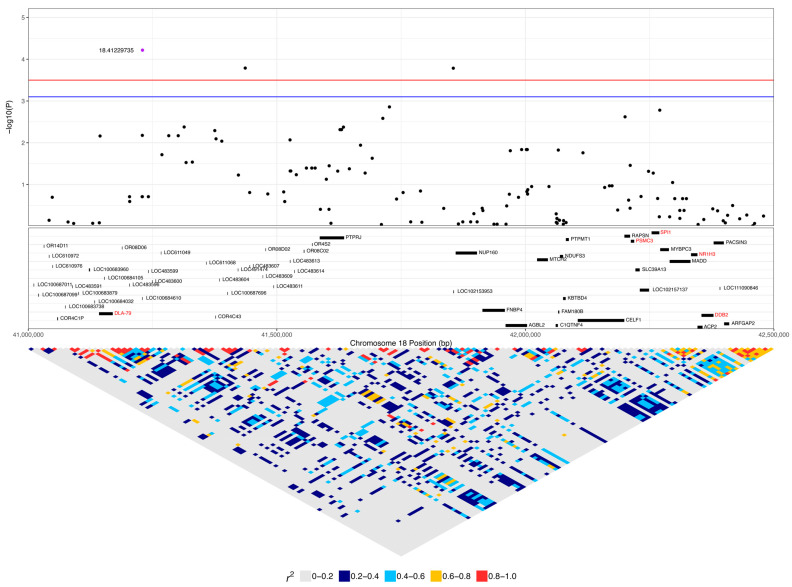
Regional association plot for a region of interest on chromosome 18. The top of the plot indicates −log_10_ *p*-values for each SNP from the mixed linear model association analysis. Red and blue lines indicate q-value cut offs of 0.05 and 0.1, respectively. The top associated SNP in this region is highlighted (purple dot). The protein-coding genes (CanFam3.1, assembly GCF_000002285.3) in the region, fetched from the NCBI, are shown in the middle box, with viral infection or cancer-associated genes identified through KEGG pathways highlighted in red. Linkage disequilibrium (r^2^) in the region is shown in the bottom triangle.

Another significant SNP, 18:41229735, had low to strong linkage (0.25 < r^2^ < 0.65) to nine SNPs (Figure 5, Table 1). This SNP had a higher frequency of homozygosity for the minor allele in lymphoma cases compared to carriers and controls (0.45, 0.22, and 0.13, respectively), while the frequency of heterozygosity was slightly greater in carriers and controls (0.56 and 0.49) compared to lymphoma cases (0.42) (Table 1). The 14 lymphoma cases that were homozygous for the minor allele included nine confirmed B-cell cases, and the age of first diagnosis averaged 9.7 years (range: 3 to 15.25 years). Only six of these cases had available staging for their lymphoma, with four cases diagnosed at stage III lymphoma and two diagnosed at stage I. Four cases were in complete remission, five cases were still undergoing chemotherapy treatment, one had chemotherapy and had had a recurrence of lymphoma, two had elected palliative care, and treatment and response were unknown for two dogs. This SNP is in exon 2 of the olfactory receptor gene *LOC100684610* (*OR4C5*) and is annotated as a missense variant (A > G), causing K249E with a SIFT value of 1 (low confidence), suggesting that the variant is tolerated (Figure 7). One of the linked SNPs, 18:41144389 (r^2^ = 0.25), is in exon 3 of *DLA-79*, and is annotated as a missense variant (C > A), causing P366T with a SIFT value of 0.01. Of the 217 sample genotypes from [27], the SNP 18:41144389 had a minor allele frequency of 0.1, and two out of seven Border Collies in their dataset carried the heterozygous genotype CA. In our population, this SNP was homozygous AA in two cases, one carrier, and two controls and heterozygous AC in 12 cases, seven carriers, and 22 controls, with an overall minor allele frequency that was also 0.1. 

The *DLA-79* gene is an MHC class Ib gene and is listed in the KEGG viral infection pathways for the human papillomavirus (HPV), Epstein–Barr virus (EBV), Kaposi sarcoma-associated herpesvirus (KSHV), human cytomegalovirus (HCMV), human immunodeficiency virus 1 (HIV-1), and human T-cell leukaemia virus 1 (HTLV-1) and in the viral carcinogenesis pathway. Chromosome 27 was significant for 29 SNPs, spanning 1.5 to 8.9 Mb (Figure 4, Table 2). This included seven SNPs within 3.1 to 3.6 Mb, nine SNPs within 5.1 to 5.8 Mb, and seven SNPs within 8.3 to 8.9 Mb. Three SNPs were within *ANO6*, two SNPs each were in *SCN8A* and *LOC111092890* (lncRNA), and there was one SNP each in *LOC486504* (pseudogene), *TARBP2*, *LOC111092799* (lncRNA), 2632 *ANKRD33*, *FIGNL2*, *BIN2*, *SPATS2*, *LOC111092735*, *LMBR1L*, *RHEBL1*, *WNT10B*, and *CCNT1*.

**Figure 8:**
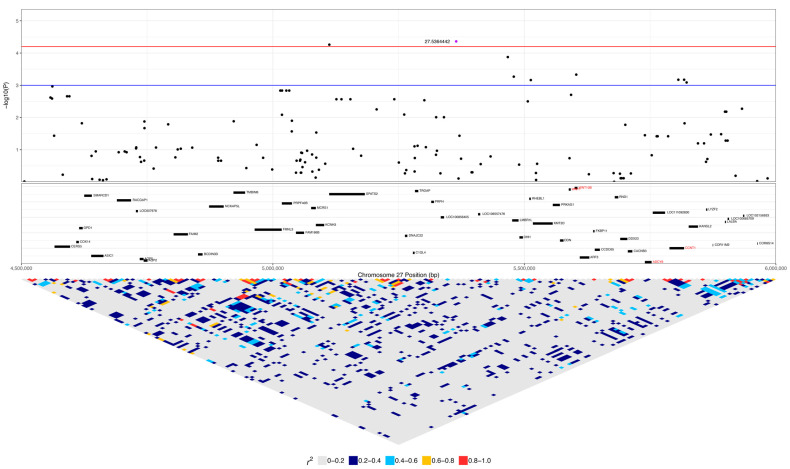
Regional association plot for the region of interest on chromosome 27. The top of the plot indicates −log_10_ *p*-values for each SNP from the mixed linear model association analysis. Red and blue lines indicate q-value cut offs of 0.05 and 0.1, respectively. The top associated SNP in this region is highlighted (purple dot). The protein-coding genes (CanFam3.1, assembly GCF_000002285.3) in the region, fetched from the NCBI, are shown in the middle box, with viral infection or cancer-associated genes identified through KEGG pathways highlighted in red. Linkage disequilibrium (r^2^) in the region is shown in the bottom triangle.

**Figure 9:**
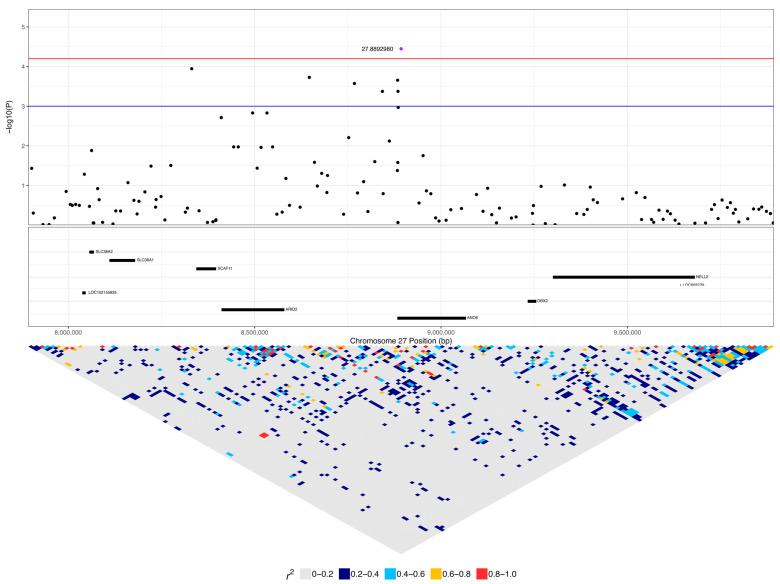
Regional association plot for the most significant region on chromosome 27. The top of the plot indicates −log_10_ *p*-values for each SNP from the mixed linear model association analysis. Red and blue lines indicate q-value cut offs of 0.05 and 0.1, respectively. The top associated SNP in this region is highlighted (purple dot). The protein-coding genes (CanFam3.1, assembly GCF_000002285.3) in the region, fetched from the NCBI, are shown in the middle box, with viral infection or cancer-associated genes identified through KEGG pathways highlighted in red. Linkage disequilibrium (r^2^) in the region is shown in the bottom triangle.

The expanded 1 to 9.4 Mb region consisted of 190 protein-coding genes, including 12 genes that were listed in cancer pathways from KEGG. These were: *SP1, ESPL1*, *ITGB7*, and *EIF4B* at 1.8 to 2.2 Mb (Supplementary Figure S1); *ATF1* at 3.9 Mb (Supplementary Figure S2); *WNT1*, *WNT10B*, *ADCY6*, and *CCNT1* at 5.6 to 5.8 Mb (Figure 8); and *PFKM*, *COL2A1*, and *HDAC7* at 6.6 to 7 Mb (Supplementary Figure S3). The top SNP on chromosome 27, 27:8892980, was in *ANO6* and had low to moderate linkage to four other SNPs on *ANO6* (0.2 < r^2^ < 0.5), which included the two other SNPs that were significant in this gene (27:8883501 and 27:8884575) (Figure 9). A total of 16% (five dogs) of lymphoma cases were homozygous for the minor allele at this top SNP, while 4% of carriers and 4% of controls were homozygous (Table 2). *ANO6* has two missense mutations annotated, both of which are predicted to not be tolerated (27:8995542 C > T, causing E14K; 27:8995524 C > T, causing E20K; SIFT = 0). There are also two in-frame deletions at 27:8995479–8995496 (causing F29_G35delinsC) and 27:89954802645–8995491 (causing F31_F34del), and there are deletions at 27:8995478–8995479 (causing G35X), 27:8995478 (causing G35X), 27:8995479–8995497 (causing F29_G35delinsX), and 27:8995480–8995492 (causing F31_F34delinsX). Another significant SNP, 27:5603116, was in an intronic region of the *WNT10B* gene, which is involved in the pathways ‘human papillomavirus infection’, ‘proteoglycans in cancer’, and ‘pathways in cancer’. This SNP was strongly linked (r^2^ > 0.8) to one SNP in a non-coding region and had low to moderate linkage (0.2 < r^2^ < 0.5) to three other SNPs, each in a different gene: *LMBR1L*, *KMT2D*, and *CACNB3* (Figure 9). This SNP was homozygous in 42% of lymphoma cases, 26% of carriers, and 5% of controls (Table 2). Carriers and controls had similar frequencies of heterozygosity at this SNP at 48% and 52%, respectively, whereas lymphoma cases had a heterozygosity frequency of 35% at this SNP. The 13 lymphoma cases that were homozygous for the minor allele included nine confirmed B-cell cases and an affected dam–offspring duo (PBC101 and PBC102) while the age of first diagnosis averaged 8.6 years (range: 1.9 to 13.3 years). Among these dogs, four went into complete remission following chemotherapy, two had partial remission and survived less than a year following the commencement of treatment, three were still undergoing treatment at the time of sampling, three underwent palliative treatment, and one was euthanised due to an advanced stage of lymphoma. *WNT10B* only has one known missense variant at 27:5604484 (C > G, causing R303G) but is predicted to be tolerated (SIFT = 0.28). *LMBR1L* has a missense variant at 27:5487906 (C > T, causing R484W in transcript ENSCAFT00000013794.4 and R458W in transcript ENSCAFT00000068147.1) that is not tolerated (SIFT = 0.01). There was also a SNP in this gene that was significant (27:5478927), and the homozygosity of the minor allele at this SNP was similar between cases (23%) and carriers (22%), but greater in these than in controls (3%). *KMT2D* has many known missense variants, in-frame deletions and insertions, frameshift mutations, and premature stop codon mutations (SIFT scores unavailable). 

A backwards stepwise regression of the top 100 SNPs was conducted to find the best combinations of SNPs to discriminate between cases and controls. A total of 74 SNPs were retained across 20 chromosomes (residual standard error (RSE) = 0.1013 on 16 degrees of freedom (DF), adjusted R^2^ = 0.9379, *p* = 3.79 × 10^−8^) (Supplementary Table S2). This included 19 SNPs from chromosome 18 and 16 SNPs from chromosome 27. Haplotype analyses were conducted for the significant regions on chromosomes 18 and 27. There were 18 haplotype blocks that were highly significant (*p* < 0.0001) on chromosome 18, ranging from 2 to 14 SNPs in size (Table 3). The top haplotype was a 12 SNP block from 18:38038090 to 18:38129762, which spanned seven olfactory genes, and was homozygous in two cases (dogs PBC098 and PBC158) and was not homozygous in any carriers or controls. A majority of the other significant haplotypes also covered olfactory genes: *LOC106559997*, *OR5B21*, *OR10C10*, *LOC483503*, *OR04E05*, *LOC483541*, and *LOC100685982*. Two haplotypes (3 SNPs and 5 SNPs in size) each had one SNP within *CTNND1*, which was homozygous only in two cases (PBC098 and PBC158) and not in any carriers or controls. Both dogs had confirmed B-cell lymphoma and entered complete remission following chemotherapy. PBC098 was diagnosed at 3 years with unknown staging, grading, or subtype, while PBC158 was diagnosed at 11 years and 5 months with stage III, high-grade, multicentric lymphoma. There were three significant SNPs and a 5 SNP haplotype spanning *ZDHHC5* that was homozygous in three cases (PBC098, PBC099, and PBC158) and not homozygous in any carriers or controls. PBC099 had also had B-cell lymphoma diagnosed at 9 years and entered complete remission following chemotherapy.

On chromosome 27, there were 29 highly significant (*p* < 0.0001) haplotype blocks, ranging from 2 to 19 SNPs in size (Table 4). The top haplotype was 9 SNPs long (from 27:5104085 to 27:5241391) and spanned the *SPATS2* gene. This haplotype was homozygous in 29% of cases (nine dogs), 7% of carriers (two dogs), and 2% of controls (two dogs). The nine dogs that were homozygous for this haplotype included four dogs diagnosed at 4 years old or younger, three of which entered complete remission following therapy while one was euthanised. Five cases were diagnosed at 9 years or older; three of these were still undergoing chemotherapy treatment at the time of sampling, one was undergoing palliative treatment, and one was euthanised. 

**Figure 10:**
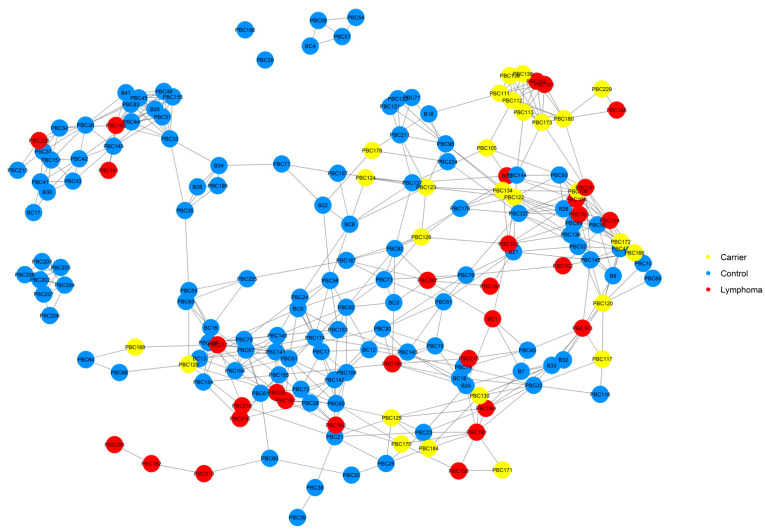
NetView relationship network of genotyped lymphoma cases (N = 31), carriers (N = 27), and controls (N = 119) based on a distance matrix. Clustered based on a *k*-value of 10.

A NetView plot was also created based on a distance matrix using the genotype data to visualise the relationships between cases, carriers, and control dogs (Figure 10). There were two small groups of controls and two individual control dogs that did not map to the main network. Three cases were part of a subcluster of the main network (Figure 10, top left) which lacked carrier dogs. These three cases had no pedigree information. An inspection of the control dogs in this cluster in the in-house pedigree database showed that this cluster primarily consisted of working Border Collies. There were eight carriers that clustered closely with three cases, which included an affected dam–daughter duo (PBC101 and PBC102) (Figure 10, top right). The other lymphoma cases were mostly spread out through the lower half and right side of the main network. 

### 3.3. GWAS with Binary Phenotype 

**Figure 11:**
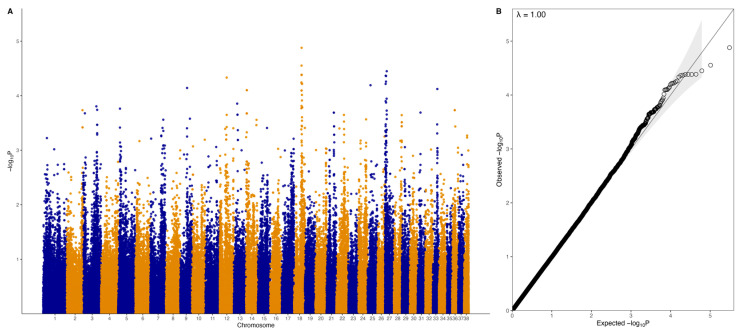
Genome-wide association results for the binary phenotype (31 lymphoma cases, 119 controls). (**A**) Manhattan plot of −log_10_ *p*-values from mixed linear model association analysis. (**B**) QQplot of expected and observed −log_10_ *p*-values; shaded area indicates 95% confidence interval.

**Figure 12:**
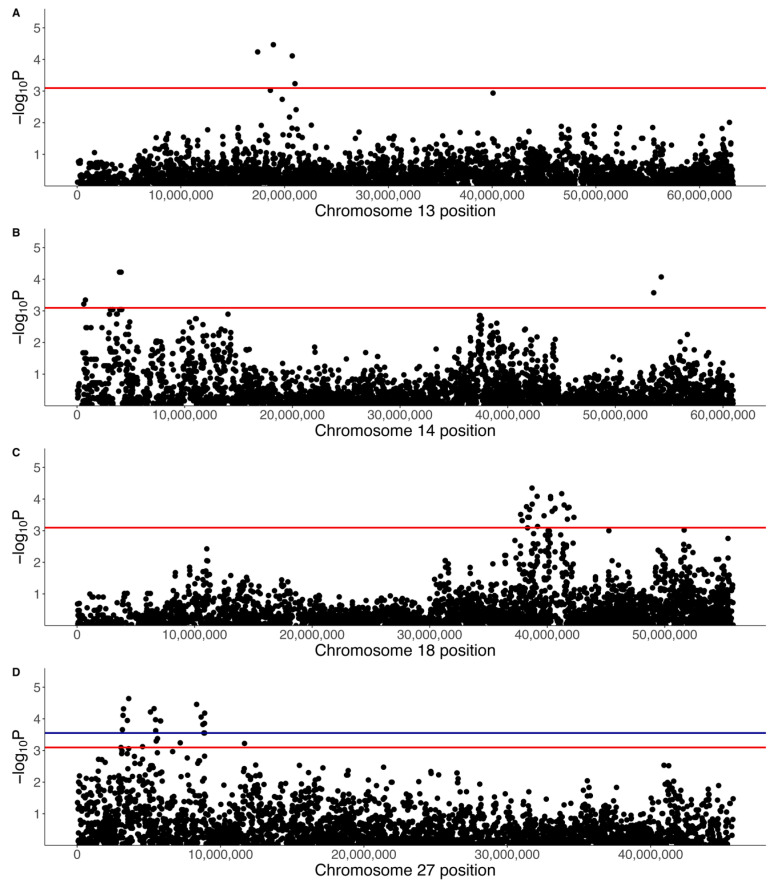
Regional Manhattan plots for the GWAS results using a binary phenotype on (**A**) chromosome 13, (**B**) chromosome 14, (**C**) chromosome 18, and (**D**) chromosome 27. The blue line indicates a chromosome q-value cut-off of 0.05 and the red line indicates a q-value cut-off of 0.1.

A separate GWAS was run with just 31 cases and 119 controls using a binary phenotype (1 for affected, 0 for control). The top SNPs did not reach genome-wide significance (Figure 11), but chromosome-wise calculated q-values showed that there were four significant SNPs on chromosome 13 (0.05 < q < 0.1), three significant SNPs on chromosome 14 (0.05 < q < 0.1), 24 significant SNPs on chromosome 18 (0.05 < q < 0.1), and 25 significant SNPs on chromosome 27 (19 SNPs with q < 0.05, six SNPs with 0.05 < q < 0.1) (Figure 12, Table 5). The significant regions on chromosomes 18 (37.7 Mb to 42.3 Mb) and 27 (3.1 Mb to 11.7 Mb) overlapped with the regions identified in the quantitative phenotype GWAS such that 21 SNPs on chromosome 18 and 23 SNPs on chromosome 27 were significant in both analyses. There were three SNPs that were significant on chromosome 18 at 41.7 Mb to 42.3 Mb that were not significant in the quantitative phenotype GWAS, none of which were located within genes, while there were two SNPs that were significant on chromosome 27 and were not significant in the quantitative phenotype GWAS at 4,561,667 bp (within *LIMA1*) and 11,672,173 bp (within the lncRNA *LOC106557903*). 

The significant SNPs on chromosome 13 spanned 17.4 to 20.7 Mb and included two SNPs within *DEPTOR*, one SNP within *EXT1,* and one SNP within the lncRNA *LOC111098540* (Table 5). There were no KEGG pathway viral infection or cancer pathway genes within 500 kb upstream and downstream of this region. The significant SNPs on chromosome 14 spanned two SNPs at 3.9 to 4.1 Mb, which included one SNP, within *EXOC4*, and one SNP at 54,260,890 bp that was not within a gene (Table 5). The expanded region 500 kb upstream and downstream of 3.9 to 4.1 Mb did not contain any KEGG viral infection or cancer pathway genes, and nor did a 500 kb region around the 14:54260890 SNP. 

A backwards stepwise regression of the top 100 SNPs was also conducted as per the quantitative GWAS to discriminate between cases and controls (Supplementary Table S3). A total of 79 SNPs remained across 21 chromosomes (RSE = 0.1186 on 3 DF, adjusted R2 = 0.9379, *p* = 3.79 × 10^−8^) (Supplementary Table S2). This included 19 SNPs from chromosome 18 and 19 SNPs from chromosome 27. Among these 79 SNPs, 54 were in common with the 74 SNPs that remained after the backwards stepwise regression of the quantitative phenotype GWAS. 

Haplotype block association analysis for the significant chromosomes, as detected in the above analysis, found chromosome 18 to have five highly significant blocks (*p* < 0.0001), ranging from 2 to 14 SNPs in size (Table 6). The 14 SNP haplotype from 18:38233567 to 18:38383741 was the most significant (*p* = 7.66 × 10^−5^ ) for this chromosome, occurring only in two cases and no controls, and spanned several olfactory genes. Chromosome 27 had seven highly significant haplotypes ranging from 2 to 12 SNPs in size (Table 7). The most significant (*p* = 2.82 × 10^−6^) on this chromosome was an 8 SNP haplotype from 27:5112245 to 27:5241391, overlapping with the most significant haplotype block in chromosome 27 in the quantitative phenotype GWAS, and occurred in nine cases (29% of cases) and two controls (2% of controls) and spanned the gene *SPATS2*. Notably, there was a 2 SNP haplotype (CA at 27:5592820 to 27:5603116) spanning the *WNT10B* gene that was homozygous in 42% of cases (13 dogs) and 5% of controls (6 dogs). There were no highly significant haplotype blocks for chromosomes 13 and 14 (*p* > 0.0001). 

### 3.4. Phenotypic Variance Explained by Genetic Variance (Heritability) 

REML analyses were conducted to estimate the phenotypic variance explained by genetic variance. A total of 12 chromosomes were significant (*p* < 0.05), and the analysis with all autosomes was also highly significant (*p* = 1.43 × 10^−6^) (Table 8). Even at the lowest prevalence tested (0.025), all autosomes explained 106.09% (standard error (SE) ± 21.77%) of the disease liability. Chromosome 27 was the most significant (*p* = 1.34 × 10^−6^) and explained at least 43.19% (±11.94%) of the disease liability at the lowest prevalence and up to 65.3% (±18.06%) at the highest prevalence (0.1). The significant region on chromosome 27 (1 to 9 Mb) explained less liability, at a minimum of 27.71% (±10.18%). Chromosome 18 was also highly significant (*p* = 1.21 × 10^−3^), explaining a minimum of 33.17% (±13.54%) at the lowest prevalence. The significant region on chromosome 18 (37 to 56 Mb) explained more disease liability than all SNPs on chromosome 18, from a minimum of 43.4% (±12.83%) up to 65.63% (±19.4%). Unexpectedly, chromosome 21 explained the most variance (V(G)/Vp = 47.43% (±13.44%), V(G)/Vp_L = 76.08% (±21.57%), *p* = 0.002). 

## 4. Discussion

Lymphoma is a common cause of mortality in dogs and has been underinvestigated compared to human lymphoma. In this study, pedigree investigations were expanded from our previous study [37] and showed further evidence that there is likely an underlying heritable genetic component to lymphoma as 54 cases were traced to a common ancestor. In the genetic investigations, a quantitative phenotype GWAS was designed that maximised the use of dogs that had known relationships to lymphoma cases by including a “carrier” group based on the assumption that a genetic risk for lymphoma is inherited as a recessive trait. Here, we identify five candidate genes that warrant further investigation: *DLA-79*, *WNT10B*, *LMBR1L*, *KMT2D*, and *CCNT1*. We also tested a binary-phenotype GWAS using cases and controls only and found a large overlap in significant regions with the quantitative phenotype GWAS (21 SNPs on chromosome 18 and 23 SNPs on chromosome 27 were significant in both analyses). 

Previous studies have investigated the pedigrees of lymphoma-affected dogs in small family groups with between three and nine cases [63,64]. In the present study, we mapped a much larger pedigree for 54 cases and were able to identify several occurrences of first- or second-degree relatives of lymphoma cases that were also diagnosed with lymphoma, including two pairs of affected full siblings, one affected aunt–niece pair, one affected uncle–niece pair, and five affected parent–offspring pairs, suggesting a likely heritable risk. There was further evidence that ancestors were possible carriers of a risk allele, such as the two full-sibling males (dog_44286 and dog_44277) that gave rise to four and twelve cases, respectively, which included three pairs of parent–offspring cases and one aunt–niece occurrence of lymphoma. The common ancestor for all 54 cases, dog_811, was also identified as the common ancestor for many dogs carrying the trapped neutrophil syndrome (TNS) mutation and the neuronal ceroid lipofuscinosis (NCL) mutation in our previous study on Border Collie diversity [30]. Dog_811 was identified as the fourth top-contributing ancestor to the population of dogs born between 2005 and 2015 [30], and so it may appear as a common ancestor for lymphoma simply because its descendants were frequently bred and made substantial contributions to the present generation of Border Collies. However, the familial occurrence of the disease still suggests a genetic risk is being transmitted. 

It should be noted that retrospective studies are often limited by the available health information on individuals in the study. It is likely that healthy, lymphoma-unaffected dogs in our pedigree were not captured in our health survey, and with no information on their ages or causes of death, the accuracy of heritability or prevalence estimations of the disease are affected. For example, the NetView plot of the genotyped dogs showed that there were three cases that had no pedigree information in a subcluster of the main network that had no carriers. This may mean that there is another population of Border Collies carrying a risk genotype for lymphoma that was not captured in our pedigree data, but it will require the identification and genotyping of more carrier dogs for this to be elucidated. 

The quantitative phenotype GWAS had the advantage of utilising known relatives of lymphoma cases as possible carriers of a genetic risk for lymphoma and identified potential candidate genes with known or predicted associations with lymphoma. The top SNP on chromosome 18 was in a non-coding region but was in low to moderate linkage (0.2 < r^2^ < 0.5) to SNPs in genes that have shown involvement in other cancers but not lymphoma, including *SLC43A1* in prostate cancer [65] and leukaemia [66]; *SLC43A3* in angiosarcoma [67]; and *SSRP1* (which also had a significant SNP) in colorectal cancer and glioma [68,69]. SSRP1 is a component of the Facilitates Chromatin Transcription (FACT) complex, which plays a role in transcriptional regulation and DNA damage repair and can also accelerate tumour transformation [70]. There are also microRNAs that regulate *SSRP1* and have been shown to promote cancer progression or malignancy [71]. Given its broad cancer biology role, it may be considered a candidate in canine lymphoma; however, only two cases were homozygous for the minor allele at the significant SNP and were homozygous for a 3 SNP haplotype spanning this gene. 

There was a significant SNP (18:41229735) within chromosome 18 within the olfactory receptor *OR4C5*, where the homozygous minor allele genotype (AA) was more common in lymphoma cases (45%) compared to carriers (22%) and controls (13%). Dogs have approximately three times as many olfactory genes as humans, but none of the genes have been studied for any involvement in cancers. This significant SNP was linked (r^2^ = 0.25) to a SNP in exon 3 of DLA-79 that is annotated as a missense variant and predicted to not be tolerated. *DLA-79* is a Major Histocompatibility Complex (MHC) class Ib gene and is thought to play a special role in the immune response, likely binding a distinct set of peptides or ligands [72]. *DLA-79* has 64% amino acid identity with the consensus sequence of *HLA-A*, *-B,* and *-C*. Risk haplotypes have been identified for human non-Hodgkin’s lymphoma at *HLA-A*, *-B,* and *-C*, and lower expression levels of *HLA-G* have been associated with aggressive lymphoma and poor prognosis [73,74,75]. 

There were also significant SNPs on chromosome 18 in the genes *ZDHHC5*, *CTNND1*, and *TMEM109*. *ZDHHC5* has been implicated in other cancers [76], while *TMEM109* is involved in regulating apoptosis [77]. *CTNND1* is a member of the catenin family and is involved with cell adhesion and Rho GTPase activity. It has been implicated in the SRC-family kinase-mediated transformation of cells [78]. Reduced expression has been shown in activated B-cell diffuse large B-cell lymphoma (ABC DLBCL), and variation in sequence or expression is associated with other cancers [79,80]. *CTNND1* may have a genetic variant in Border Collies that has influenced the expression of the gene or increases the risk for lymphoma; however, only two cases shared homozygous haplotypes across the gene. 

On chromosome 27, three significant SNPs (including the top SNP) were within *ANO6* (anoctamin 6). There has yet to be evidence that the gene is involved in lymphoma, but it has shown a role in cellular apoptosis through pyroptosis and ferroptosis [81,82]. The associated region on this chromosome was large (7–8Mb in size) and included 12 genes listed in cancer pathways: *SP1*, *ESPL1*, *ITGB7*, *EIF4B*, *ATF1*, *WNT1*, *WNT10B*, *ADCY6*, *CCNT1*, *PFKM*, *COL2A1*, and *HDAC7*. Among these genes, only *WNT10B* and *CCNT1* harboured a significant SNP. There were also significant haplotypes that had SNPs spanning *CCNT1* (5 SNP haplotype) and *PFKM* (4 SNP haplotype), both of which were more common in lymphoma cases (13–19%) compared to controls (13%). 

For the significant SNP in *WNT10B*, there was a higher frequency of lymphoma cases (42%) that were homozygous for the minor allele than carriers (26%) and controls (5%). *WNT10B* is directly downstream of the cancer pathway gene *WNT1* (~8 kb away). *WNT10B* and *WNT1* are members of the Wnt signalling pathway, which plays a crucial role in development, the regulation of stem cells, and tissue homeostasis [83,84]. The Wnt pathway is implicated in multiple cancers and has been shown to be activated in DLBCL [83,85,86]. Other Wnt genes have been implicated in haematopoietic malignancies, such as *WNT5A,* which has been identified as a tumour suppressor that inhibits B-cell proliferation and is a motility factor in Hodgkin’s lymphoma cells [87,88]. *WNT10B* is associated with multiple cancers and an intronless variant has been found in acute myeloid leukaemia [89,90], but the gene has not yet shown involvement in lymphoma. This region could be explored for possible functional variants associated with canine lymphoma risk given the involvement of the Wnt pathway in numerous cancers including haematopoietic malignancies. 

The significant *WNT10B* SNP was also linked (0.2 < r^2^ < 0.5) to one SNP each in *CACNB3*, *LMBR1L*, and *KMT2D*. *CACNB3* encodes a calcium voltage-gated channel and has no known role in cancer. *LMBR1L* (limb development membrane protein 1 like) has an essential role in lymphopoiesis and lymphoid activation [91]. There was one significant SNP in *LMBR1L* and there is one known missense variant (27:5487906) in this gene that is not tolerated. *LMBR1L* should therefore be explored further since mutations in this gene have the potential to affect normal lymphocyte development. 

*KMT2D* (lysine methyltransferase 2D) is a widely studied gene in the cancer context. Multiple mutations and abnormal expression of this tumour suppressor gene and epigenetic modifier have been associated with DLBCL, follicular lymphoma, T-cell lymphomas, and several other cancers [92,93,94,95,96,97,98]. Notably, one study found that the treatment of T-lymphoma cells with the histone deacetylase inhibitor chidamide and the hypomethylating agent decitabine induced apoptosis and stopped tumour growth by increasing the interaction between *KMT2D* and *SPI1* (*PU.1*) [99], which was in the associated region on chromosome 18 in the present study. The fact that it was within a significant region, and has numerous known mutations, would suggest that *KMT2D* may harbour a risk genotype for canine lymphoma. 

There was one significant SNP in *CCNT1* that had a greater frequency of carriers homozygous for the minor allele (22%) compared to cases (13%) and controls (1%). The 5 SNP haplotype that included SNPs in *CCNT1* was also homozygous in the same proportion for each group. CCNT1 forms a complex with the CDK9 serine/threonine kinase, and together, CDK9/CCNT1 is required for the differentiation of several cell types, including the differentiation and activation of lymphoid cells [100,101]. An imbalance of expression levels between CDK9 and CCNT1 has been found in several lymphoma types, suggesting a common mechanism of deregulation of transcription in the neoplastic transformation of these cells [100]. If this gene harbours a risk genotype for lymphoma, it is not clear why carriers have a higher proportion of homozygotes compared to cases. It is possible that the “carriers” identified in this study may develop lymphoma later in their life or that this is one factor among a combination of genetic factors necessary to cause lymphoma. The involvement of this gene in lymphoid cells and its expression in several lymphomas make it a good candidate for further investigation for a risk genotype for canine lymphoma. 

A significant 4 SNP haplotype spanned the cancer pathway gene *PFKM* (phosphofructokinase, muscle)*,* and homozygosity for this haplotype was more common in cases (19%) compared to carriers (7%) and controls (3%). PFKM is part of the glycolysis pathway, and posttranslational modifications of the enzyme and silencing by miRNA can promote cancer cell proliferation or adaptation to metabolic stress [102,103]. However, there has yet to be evidence of direct involvement in lymphoma. 

The results from the binary-phenotype GWAS almost completely overlapped with the results from the quantitative phenotype, with 21 SNPs on chromosome 18 and 23 SNPs on chromosome 27 being significant in both analyses. There were additional chromosome-wise significant associations on chromosomes 13 and 14, which included SNPs within the genes *EXT1*, *DEPTOR*, and *EXOC4*. *EXT1* (exostosin-1) encodes a glycosyltransferase that is required in heparan sulphate biosynthesis. Heparan sulphate is a linear polysaccharide that is found ubiquitously as a proteoglycan and is involved in a wide range of biological processes such as embryonic development, metabolism, and cell signalling, which consequently means that defects in its synthesis causes many different diseases [104]. *EXT1* is a tumour suppressor gene that has downregulated expression in acute lymphoblastic leukaemia and is frequently hypermethylated in leukaemia, causing the loss of heparan sulphate synthesis [105]. Given the broad range of effects of *EXT1* through heparan sulphate biosynthesis and its role as a tumour suppressor, it is possible this gene could be involved in lymphoma, but this has yet to be reported. 

*DEPTOR* (DEP domain-containing mTOR interacting protein) is another tumour suppressor gene and is an important regulator of *mTOR* [106]. Mechanistic target of rapamycin (*mTOR*) is a protein kinase that regulates a range of processes important in cancer, such as cell proliferation and autophagy, and many inhibitors of *mTOR* have been developed for cancer treatment [107]. Downregulation of *DEPTOR* has been shown to induce apoptosis and increase sensitivity to doxorubicin in human multiple myeloma cells [108]. Doxorubicin is commonly used in chemotherapy protocols for the treatment of canine lymphoma [109], but whether *DEPTOR* affects sensitivity to doxorubicin in lymphoma cells is not known. *DEPTOR* has also been found to regulate migration and cytokine expression in DLBCL cells through its interaction with microRNA-155 (MiR-155) [110]. MiR-155 is one of the most widely studied microRNAs, particularly in lymphoma. It is considered a biomarker for B-cell malignancies, it can increase lymphoma cell motility, and its expression levels have been correlated with prognosis in B-cell lymphoma [111,112,113]. MiR155 can also inhibit the transcription factor *SPI1*, which was within the associated region in chromosome 18 in the present study, and the upregulation of MiR-155 and downregulation of *SPI1* have been observed in numerous lymphoma types [114]. Genetic variants in *DEPTOR* may affect the interaction with MiR-155 and suggest an avenue for further investigation. 

*EXOC4* (exocyst complex component 4; also known as *SEC8*) encodes a component of the exocyst complex that has roles in cell migration [115]. One study examined malignant peripheral sheath tumour cells and found reduced levels of EXOC4 after treatment with doxorubicin and sorafenib, reporting that EXOC4 was involved in the regulation of Bcl-2 [116]. *Bcl-2* is one of the most widely studied genes in haematological malignancies, especially in diffuse large B-cell lymphoma (DLBCL) [117,118]. *EXOC4* does not currently have a clear role in lymphoma, but it may be involved by sensitising lymphoma cells to doxorubicin or affecting Bcl-2 transcription. 

Chromosome 21 explained the greatest variance in disease liability when all SNPs were included in the analyses, while significant regions across chromosomes 18 (37–56 Mb) and 27 (1–9 Mb) explained a considerable proportion of disease liability, even at the lowest prevalence levels tested—43.4% (±12.83%) and 27.71% (±10.18%), respectively. These estimates are higher than those from a previous GWAS on hemangiosarcoma and B-cell lymphoma in Golden Retrievers, which found a region on chromosome 5 (25–40 Mb) that harboured two shared risk loci for both cancers, explaining 22.4% (±10.7%) of variance [22]. The study also estimated the variance explained by the region for B-cell lymphoma only and found that it explained 60% (±25%) at a prevalence of 0.0625 [22]. At the closest prevalence in our study (0.05), the chromosome 18 region explained a similar amount of variance, at 52.93% (±15.65%), albeit across a slightly larger region (19 Mb compared to 15 Mb). The chromosome 27 region in our study explained 33.79% (±12.42%) at a prevalence of 0.05 although this region was only 8 Mb in size. 

The GWAS findings in the current study differ from those of previous GWAS on canine lymphoma [22,23,24,35]. Including the present study, each GWAS on canine lymphoma has now identified associated regions on different chromosomes (Chr 2, 4, 5, 8, 11, 14, 18, 20, and 27). This suggests that there are distinct genetic mechanisms underlying the risk of developing lymphoma in certain breeds or breed clades or that common oncogenic pathways are influenced by variants with different frequencies in different breeds. This is supported by a multi-breed multi-cancer study that identified non-coding sequence variants associated with haematopoietic malignancies [35]. 

The main limitations to this study were the lack of reported details on each lymphoma case. Nearly half of the cases had no immunotype diagnosis, which is common for older cases where palliative care is elected. Most of the genotyped cases were diagnosed as older dogs, and so, parents were mostly deceased or were not available for genotyping. Only four of the youngest cases (≤5 years) had pedigrees available, and only two sets of trios were available for genotyping. Prospective studies including more relatives could explore family-based association testing and, preferably, sequence data, which would increase the chance of sampling rare variants. It could also allow the use of alternate methods, such as the genome-wide association study by proxy (GWAX) method, which utilises first-degree relatives [119]. Lastly, due to our limited sample size, a relaxed q-value cut-off of 0.1 was used to detect more associations that could be biologically relevant; however, this would have increased the risk of false positive associations. 

In conclusion, despite limitations in sample size due to opportunistic sampling, pedigree analyses showed evidence of a potential heritable risk of lymphoma, with nine pairs involving an affected parent—offspring, littermates, and an uncle/aunt—niece identified, and 54 cases could be traced to a common ancestor. The pedigree relationships were used to design a GWAS that identified genomic regions and associated genes that may be considered candidates for lymphoma risk in Border Collies. The results from this approach support the case-control GWAS, indicating that the inclusion of putative carriers will be useful for future canine studies. 

## Figures and Tables

**Table 1:** Frequency of homozygosity and heterozygosity in lymphoma cases (N = 31), carriers (N = 27), and controls (N = 119) for significant SNPs (q < 0.1) on chromosome 18. CanFam3.1 (assembly GCF_000002285.3) genes were fetched from NCBI. A1 = minor allele; A2 = major allele; Freq = frequency; b = beta-coefficient; SE = standard error; Lym = lymphoma; Carr = carriers; Cont = controls.

Location (Chr:BP)	*p*-Value	q-Value	A1	A2	A1 Freq	b	SE	Gene	Frequency of A1A1 (No. of Dogs)			Frequency of A1A2 (No. of Dogs)		
									Lym.	Carr.	Cont.	Lym.	Carr.	Cont.
18:38704682	1.32 × 10^−5^	0.022	A	G	0.32	0.36	0.08	-	0.26 (8)	0.19 (5)	0.08 (9)	0.55 (17)	0.33 (9)	0.37 (44)
18:38233567	2.80 × 10^−5^	0.022	A	G	0.12	0.55	0.13	-	0.06 (2)	0 (0)	0 (0)	0.42 (13)	0.41 (11)	0.13 (15)
18:38719709	4.12 × 10^−5^	0.022	G	A	0.32	0.34	0.08	-	0.23 (7)	0.22 (6)	0.08 (9)	0.55 (17)	0.37 (10)	0.34 (41)
18:38502268	4.15 × 10^−5^	0.022	A	G	0.14	0.50	0.12	ZDHHC5	0.1 (3)	0 (0)	0 (0)	0.39 (12)	0.41 (11)	0.16 (19)
18:38507461	4.15 × 10^−5^	0.022	A	G	0.14	0.50	0.12	ZDHHC5	0.1 (3)	0 (0)	0 (0)	0.39 (12)	0.41 (11)	0.16 (19)
18:38510335	4.15 × 10^−5^	0.022	A	G	0.14	0.50	0.12	ZDHHC5	0.1 (3)	0 (0)	0 (0)	0.39 (12)	0.41 (11)	0.16 (19)
18:37737740	5.70 × 10^−5^	0.022	C	A	0.14	0.49	0.12	-	0.06 (2)	0 (0)	0 (0)	0.45 (14)	0.41 (11)	0.16 (19)
18:39140112	5.97 × 10^−5^	0.022	G	A	0.25	0.38	0.09	LOC100688997	0.16 (5)	0.07 (2)	0.03 (4)	0.58 (18)	0.33 (9)	0.34 (41)
18:41229735	6.06 × 10^−5^	0.022	A	G	0.44	0.33	0.08	LOC100684610 (OR4C5)	0.45 (14)	0.22 (6)	0.13 (15)	0.42 (13)	0.56 (15)	0.49 (58)
18:38350947	8.02 × 10^−5^	0.023	A	G	0.12	0.51	0.13	-	0.06 (2)	0 (0)	0 (0)	0.42 (13)	0.41 (11)	0.13 (16)
18:38456518	8.02 × 10^−5^	0.023	A	G	0.12	0.51	0.13	CTNND1	0.06 (2)	0 (0)	0 (0)	0.42 (13)	0.41 (11)	0.13 (16)
18:40281122	9.65 × 10^−5^	0.026	A	G	0.42	0.31	0.08	-	0.42 (13)	0.19 (5)	0.13 (16)	0.42 (13)	0.56 (15)	0.45 (53)
18:40286669	1.22 × 10^−4^	0.030	G	A	0.43	0.31	0.08	-	0.42 (13)	0.19 (5)	0.13 (16)	0.42 (13)	0.56 (15)	0.47 (56)
18:38326192	1.54 × 10^−4^	0.030	A	G	0.14	0.46	0.12	OR10C10	0.06 (2)	0 (0)	0 (0)	0.42 (13)	0.41 (11)	0.17 (20)
18:41436427	1.63 × 10^−4^	0.030	C	A	0.50	−0.31	0.08	-	0.1 (3)	0.07 (2)	0.35 (42)	0.42 (13)	0.67 (18)	0.45 (53)
18:41854962	1.64 × 10^−4^	0.030	C	G	0.15	0.42	0.11	-	0.1 (3)	0.04 (1)	0.01 (1)	0.32 (10)	0.44 (12)	0.17 (20)
18:37862012	1.69 × 10^−4^	0.030	A	T	0.18	0.40	0.11	-	0.19 (6)	0.04 (1)	0 (0)	0.32 (10)	0.41 (11)	0.24 (28)
18:37867871	1.69 × 10^−4^	0.030	A	G	0.18	0.40	0.11	OR5B21	0.19 (6)	0.04 (1)	0 (0)	0.32 (10)	0.41 (11)	0.24 (28)
18:40667579	2.48 × 10^−4^	0.042	C	A	0.47	0.30	0.08	-	0.45 (14)	0.22 (6)	0.15 (18)	0.45 (14)	0.56 (15)	0.5 (60)
18:40653765	2.87 × 10^−4^	0.046	A	C	0.47	0.30	0.08	-	0.45 (14)	0.22 (6)	0.15 (18)	0.45 (14)	0.56 (15)	0.53 (63)
18:39177075	3.56 × 10^−4^	0.053	A	G	0.32	0.32	0.09	-	0.23 (7)	0.15 (4)	0.08 (9)	0.55 (17)	0.44 (12)	0.38 (45)
18:40418483	3.64 × 10^−4^	0.053	A	G	0.47	0.29	0.08	-	0.42 (13)	0.22 (6)	0.16 (19)	0.48 (15)	0.52 (14)	0.52 (62)
18:55380537	3.89 × 10^−4^	0.054	A	T	0.44	−0.30	0.08	TMEM109	0 (0)	0 (0)	0.29 (35)	0.48 (15)	0.59 (16)	0.47 (56)
18:37249960	4.14 × 10^−4^	0.055	A	G	0.14	0.40	0.11	LOC483451	0.1 (3)	0 (0)	0.01 (1)	0.39 (12)	0.37 (10)	0.16 (19)
18:39727438	5.07 × 10^−4^	0.065	G	A	0.31	0.29	0.08	-	0.29 (9)	0.11 (3)	0.08 (9)	0.45 (14)	0.48 (13)	0.35 (42)
18:38838399	6.64 × 10^−4^	0.082	G	A	0.48	0.26	0.08	-	0.45 (14)	0.33 (9)	0.21 (25)	0.45 (14)	0.37 (10)	0.43 (51)
18:38807806	7.17 × 10^−4^	0.085	A	G	0.15	0.41	0.12	SSRP1	0.06 (2)	0 (0)	0 (0)	0.45 (14)	0.37 (10)	0.2 (24)
18:40076496	8.15 × 10^−4^	0.093	G	A	0.21	0.34	0.10	-	0.13 (4)	0.04 (1)	0.02 (2)	0.48 (15)	0.41 (11)	0.29 (34)

**Table 2:** Frequency of homozygosity and heterozygosity in lymphoma cases (N = 31), carriers (N = 27), and controls (N = 119) for significant SNPs (q < 0.1) on chromosome 27. CanFam3.1 (assembly GCF_000002285.3) genes were fetched from NCBI. Non-protein-coding genes are indicated in italics. A1 = minor allele; A2 = major allele; Freq = frequency; b = beta-coefficient; SE = standard error; Lym = lymphoma; Carr = carriers; Cont = controls.

Location (Chr:BP)	*p*-Value	q-Value	A1	A2	A1 Freq	b	SE	Gene	Frequency of A1A1 (No. of Dogs)			Frequency of A1A2 (No. of Dogs)		
									Lym.	Carr.	Cont.	Lym.	Carr.	Cont.
27:8892980	3.55 × 10^−5^	0.038	C	G	0.22	0.36	0.09	ANO6	0.16 (5)	0.04 (1)	0.04 (5)	0.55 (17)	0.37 (10)	0.25 (30)
27:3594560	4.29 × 10^−5^	0.038	G	A	0.15	0.42	0.10	BIN2	0.13 (4)	0.07 (2)	0 (0)	0.45 (14)	0.26 (7)	0.18 (21)
27:5364442	4.35 × 10^−5^	0.038	A	G	0.18	0.39	0.10	*LOC111092735*	0.16 (5)	0.07 (2)	0.01 (1)	0.42 (13)	0.33 (9)	0.22 (26)
27:5112245	5.50 × 10^−5^	0.038	A	G	0.24	0.34	0.09	SPATS2	0.29 (9)	0.11 (3)	0.02 (2)	0.39 (12)	0.52 (14)	0.27 (32)
27:3234647	6.07 × 10^−5^	0.038	G	A	0.18	0.39	0.10	SCN8A	0.13 (4)	0.07 (2)	0.03 (3)	0.48 (15)	0.26 (7)	0.19 (23)
27:8331252	1.13 × 10^−4^	0.053	G	A	0.12	0.44	0.11	-	0.06 (2)	0.04 (1)	0 (0)	0.45 (14)	0.26 (7)	0.13 (15)
27:3197949	1.26 × 10^−4^	0.053	G	A	0.18	0.37	0.10	SCN8A	0.13 (4)	0.07 (2)	0.03 (3)	0.48 (15)	0.26 (7)	0.21 (25)
27:5467028	1.33 × 10^−4^	0.053	G	A	0.36	0.30	0.08	-	0.32 (10)	0.11 (3)	0.09 (11)	0.52 (16)	0.48 (13)	0.41 (49)
27:8646723	1.87 × 10^−4^	0.066	G	A	0.13	0.41	0.11	-	0.06 (2)	0.04 (1)	0 (0)	0.45 (14)	0.26 (7)	0.17 (20)
27:8883501	2.21 × 10^−4^	0.070	A	G	0.15	0.37	0.10	ANO6	0.13 (4)	0.04 (1)	0.01 (1)	0.42 (13)	0.26 (7)	0.17 (20)
27:8767784	2.67 × 10^−4^	0.077	T	A	0.14	0.40	0.11	*LOC111092890*	0.06 (2)	0.04 (1)	0 (0)	0.45 (14)	0.26 (7)	0.18 (21)
27:3501246	3.35 × 10^−4^	0.082	C	A	0.37	−0.30	0.08	-	0.03 (1)	0.11 (3)	0.2 (24)	0.29 (9)	0.33 (9)	0.47 (56)
27:3054141	3.62 × 10^−4^	0.082	A	G	0.23	0.32	0.09	-	0.13 (4)	0.07 (2)	0.04 (5)	0.55 (17)	0.48 (13)	0.25 (30)
27:8842830	4.22 × 10^−4^	0.082	A	C	0.13	0.38	0.11	*LOC111092890*	0.06 (2)	0.04 (1)	0.01 (1)	0.45 (14)	0.26 (7)	0.14 (17)
27:8884575	4.22 × 10^−4^	0.082	A	G	0.13	0.38	0.11	ANO6	0.06 (2)	0.04 (1)	0.01 (1)	0.45 (14)	0.26 (7)	0.14 (17)
27:1953293	4.40 × 10^−4^	0.082	G	A	0.23	0.33	0.09	*LOC111092799*	0.13 (4)	0.07 (2)	0.03 (3)	0.52 (16)	0.48 (13)	0.3 (36)
27:3154712	4.58 × 10^−4^	0.082	A	G	0.34	0.29	0.08	FIGNL2	0.26 (8)	0.15 (4)	0.08 (10)	0.55 (17)	0.41 (11)	0.4 (48)
27:5603116	4.67 × 10^−4^	0.082	A	G	0.39	0.28	0.08	WNT10B	0.42 (13)	0.26 (7)	0.05 (6)	0.35 (11)	0.48 (13)	0.52 (62)
27:5478927	5.41 × 10^−4^	0.083	A	G	0.31	0.29	0.08	LMBR1L	0.23 (7)	0.22 (6)	0.03 (4)	0.52 (16)	0.33 (9)	0.43 (51)
27:7188905	5.43 × 10^−4^	0.083	A	G	0.28	0.30	0.09	-	0.23 (7)	0.11 (3)	0.04 (5)	0.48 (15)	0.44 (12)	0.34 (41)
27:3104073	5.47 × 10^−4^	0.083	A	G	0.23	0.31	0.09	ANKRD33	0.13 (4)	0.07 (2)	0.03 (4)	0.52 (16)	0.48 (13)	0.26 (31)
27:5806033	6.73 × 10^−4^	0.089	A	G	0.22	0.32	0.10	CCNT1	0.13 (4)	0.22 (6)	0.01 (1)	0.55 (17)	0.33 (9)	0.25 (30)
27:5817551	6.73 × 10^−4^	0.089	G	A	0.22	0.32	0.10	-	0.13 (4)	0.22 (6)	0.01 (1)	0.55 (17)	0.33 (9)	0.25 (30)
27:5512765	6.89 × 10^−4^	0.089	A	G	0.36	0.28	0.08	RHEBL1	0.29 (9)	0.11 (3)	0.08 (9)	0.52 (16)	0.48 (13)	0.47 (56)
27:6657558	7.32 × 10^−4^	0.089	G	A	0.23	0.31	0.09	-	0.19 (6)	0.07 (2)	0.03 (3)	0.45 (14)	0.37 (10)	0.31 (37)
27:6660738	7.32 × 10^−4^	0.089	A	G	0.23	0.31	0.09	-	0.19 (6)	0.07 (2)	0.03 (3)	0.45 (14)	0.37 (10)	0.31 (37)
27:1488082	7.65 × 10^−4^	0.090	G	A	0.35	0.27	0.08	*LOC486504*	0.29 (9)	0.11 (3)	0.12 (14)	0.45 (14)	0.44 (12)	0.39 (46)
27:5822515	8.18 × 10^−4^	0.093	G	A	0.22	0.31	0.09	-	0.13 (4)	0.22 (6)	0.01 (1)	0.55 (17)	0.37 (10)	0.25 (30)
27:1719544	9.13 × 10^−4^	0.100	A	G	0.34	0.27	0.08	TARBP2	0.26 (8)	0.07 (2)	0.17 (20)	0.45 (14)	0.41 (11)	0.31 (37)

**Table 3:** Frequency of homozygosity in lymphoma cases (N = 31), carriers (N = 27), and controls (N = 119) at highly significant haplotypes (*p* < 0.0001) from a linear regression of haplotype blocks for chromosome 18. Genes in the region were fetched from NCBI (CanFam3.1, assembly CF_000002285.3). Lym = lymphoma; Carr = carriers; Cont = controls.

No. of SNPs	Start SNP	End SNP	Haplotype	*p*-Value	Genes in Region	Frequency of Homozygosity (No. of Dogs)		
						Lym.	Carr.	Cont.
12	18:38038090	18:38129762	AACAAAACAACG	7.75 × 10^−8^	LOC106559970, OR08G08, LOC483477, COR5BC3, LOC483479, LOC610022, LOC483480	0.06 (2)	0 (0)	0 (0)
2	18:39123584	18:39140112	AG	1.65 × 10^−7^	LOC100688997	0.1 (3)	0.07 (2)	0.02 (2)
2	18:37724157	18:37737740	CC	5.77 × 10^−7^	LOC100686230	0.06 (2)	0 (0)	0 (0)
5	18:38383741	18:38426687	GGGAG	6.09 × 10^−7^	CTNND1	0.06 (2)	0 (0)	0 (0)
3	18:38456518	18:38478390	AAC	6.09 × 10^−7^	CTNND1, BTBD18	0.06 (2)	0 (0)	0 (0)
5	18:38498673	18:38511357	AAAAT	2.31 × 10^−6^	ZDHHC5	0.1 (3)	0 (0)	0 (0)
2	18:55380537	18:55385901	TG	3.36 × 10^−6^	TMEM109	0.52 (16)	0.41 (11)	0.24 (28)
2	18:37862012	18:37867871	AA	6.12 × 10^−6^	OR5B21	0.19 (6)	0.04 (1)	0 (0)
2	18:37862012	18:37867871	TG	6.12 × 10^−6^	OR5B21	0.48 (15)	0.56 (15)	0.76 (91)
2	18:38317284	18:38326192	AA	1.07 × 10^−5^	OR10C10	0.06 (2)	0 (0)	0 (0)
14	18:13130318	18:13325298	AGAAAAAAGAAGAG	1.27 × 10^−5^	COG5	0 (0)	0 (0)	0 (0)
3	18:41713196	18:41740841	GGG	4.05 × 10^−5^	LOC106559997	0.06 (2)	0.07 (2)	0.33 (39)
3	18:38805092	18:38807806	AGA	4.45 × 10^−5^	SSRP1	0.06 (2)	0 (0)	0 (0)
2	18:41422687	18:41436427	AC	6.03 × 10^−5^	-	0.1 (3)	0.07 (2)	0.35 (42)
3	18:39168396	18:39195972	AAA	6.86 × 10^−5^	LOC483503, OR04E05	0.1 (3)	0.04 (1)	0.01 (1)
5	18:39846804	18:39860197	AGGAA	8.23 × 10^−5^	LOC483541, LOC100685982	0.16 (5)	0.04 (1)	0.01 (1)
2	18:40663070	18:40667579	GC	8.38 × 10^−5^	-	0.45 (14)	0.22 (6)	0.15 (18)
3	18:38719709	18:38732149	GAA	8.47 × 10^−5^	SLC43A3	0.23 (7)	0.22 (6)	0.08 (9)

**Table 4:** Frequency of homozygosity in lymphoma cases (N = 31), carriers (N = 27), and controls (N = 119) at highly significant haplotypes (*p* < 0.0001) from a linear regression of haplotype blocks for chromosome 27. Genes in the region were fetched from the NCBI (CanFam3.1, assembly GCF_000002285.3). Non-protein-coding genes are indicated in italics. Lym = lymphoma; Carr = carriers; Cont = controls.

No. of SNPs	Start SNP	End SNP	Haplotype	*p*-Value	Genes in Region	Frequency of Homozygosity (No. of Dogs)		
						Lym.	Carr.	Cont.
9	27:5104085	27:5241391	AAAAGCGAA	9.37 × 10^−9^	SPATS2	0.29 (9)	0.07 (2)	0.02 (2)
6	27:4662318	27:4728424	GAAGGA	1.27 × 10^−8^	ASIC1, RACGAP1, *LOC111092823*	0.13 (4)	0.07 (2)	0 (0)
5	27:5304179	27:5340118	GAAAC	2.04 × 10^−8^	PRPH, LOC100856405	0.16 (5)	0.07 (2)	0.01 (1)
4	27:5919807	27:5983459	AGAA	8.05 × 10^−8^	cOR8S6P, LOC102156933, LOC100855426, COR8S14, *LOC111092874*	0.1 (3)	0.07 (2)	0 (0)
4	27:8010819	27:8043205	AGAA	1.11 × 10^−7^	*LOC102154451*, LOC102155835	0.06 (2)	0.04 (1)	0 (0)
5	27:3590788	27:3615721	AGAGA	1.33 × 10^−7^	BIN2, SMAGP	0.13 (4)	0.07 (2)	0 (0)
5	27:5762992	27:5817551	GAGAG	1.74 × 10^−7^	LOC111092830, CCNT1	0.13 (4)	0.22 (6)	0.01 (1)
9	27:8396219	27:8548130	AGCGCGGGG	2.27 × 10^−7^	SCAF11, ARID2	0.06 (2)	0.04 (1)	0 (0)
2	27:8314156	27:8331252	AG	2.57 × 10^−7^	*LOC111092841*	0.06 (2)	0.04 (1)	0 (0)
13	27:3197949	27:3347721	GTCGAGGAG GGGC	3.97 × 10^−7^	SCN8A	0.13 (4)	0.07 (2)	0 (0)
4	27:5919807	27:5983459	AAAA	2.70 × 10^−6^	cOR8S6P, LOC102156933, LOC100855426, COR8S14, *LOC111092874*	0.16 (5)	0.11 (3)	0.45 (53)
3	27:5261130	27:5265185	GGA	4.07 × 10^−6^	DNAJC22	0.29 (9)	0.19 (5)	0.08 (9)
4	27:8583844	27:8646723	CAAG	5.07 × 10^−6^	*LOC102155300*	0.06 (2)	0.04 (1)	0 (0)
3	27:8842830	27:8861426	AAA	8.77 × 10^−6^	*LOC111092890*	0.06 (2)	0.04 (1)	0.01 (1)
6	27:8883501	27:8885135	AGAAAA	8.77 × 10^−6^	ANO6	0.06 (2)	0.04 (1)	0.01 (1)
2	27:8767784	27:8775933	TG	1.81 × 10^−5^	*LOC111092890*	0.06 (2)	0.04 (1)	0 (0)
3	27:5281537	27:5282177	CAG	2.24 × 10^−5^	C1QL4	0.23 (7)	0.22 (6)	0.05 (6)
6	27:21881521	27:21942273	AAAGGG	2.33 × 10^−5^	*LOC111092834*	0.03 (1)	0.04 (1)	0.02 (2)
3	27:5861077	27:5870632	GGA	2.99 × 10^−5^	LYZF2	0.35 (11)	0.3 (8)	0.12 (14)
4	27:1460779	27:1471371	AAGG	3.26 × 10^−5^	*LOC486504*	0.32 (10)	0.22 (6)	0.06 (7)
3	27:1250330	27:1251390	AGA	3.96 × 10^−5^	LOC607625	0.1 (3)	0.07 (2)	0.01 (1)
5	27:4735827	27:4744789	AAGCA	3.96 × 10^−5^	AQP5, AQP2	0.35 (11)	0.22 (6)	0.09 (11)
3	27:5261130	27:5265185	AGA	4.21 × 10^−5^	DNAJC22	0.03 (1)	0 (0)	0.13 (15)
3	27:3102259	27:3104073	GAA	4.74 × 10^−5^	ANKRD33	0.13 (4)	0.07 (2)	0.03 (4)
5	27:11605144	27:11653990	CGAGA	5.17 × 10^−5^	*LOC106557903*	0.1 (3)	0.3 (8)	0.05 (6)
2	27:2965369	27:2966261	GC	5.84 × 10^−5^	-	0.87 (27)	0.74 (20)	0.53 (63)
5	27:21532742	27:21616626	GCAGC	7.16 × 10^−5^	RASSF8, LOC106557908	0.03 (1)	0.04 (1)	0.02 (2)
19	27:4373882	27:4565172	AAAGAGGAGGAGAGAAAAG	8.25 × 10^−5^	LARP4, FAM186A, LIMA1	0.13 (4)	0.07 (2)	0.03 (4)
4	27:6639027	27:6661792	CGAA	9.24 × 10^−5^	PFKM	0.19 (6)	0.07 (2)	0.03 (3)

**Table 5:** Frequency of homozygosity and heterozygosity in lymphoma cases (N = 31) and controls (N = 119) for significant SNPs (q < 0.1) in the binary phenotype GWAS. CanFam3.1 (assembly GCF_000002285.3) genes were fetched from NCBI. Non-protein-coding genes are indicated in italics. A1 = minor allele; A2 = major allele; Freq = frequency; b = beta-coefficient; SE = standard error; Lym = lymphoma; Cont = controls.

Location (Chr:BP)	*p*-Value	q-Value	A1	A2	A1 Freq	b	SE	Gene	Frequency of A1A1 (No. of Dogs)		Frequency of A1A2 (No. of Dogs)	
									Lym.	Cont.	Lym.	Cont.
13:17399337	5.78 × 10^−5^	0.087	A	C	0.02	0.63	0.16	EXT1	0 (0)	0 (0)	0.19 (6)	0.01 (1)
13:18910196	3.42 × 10^−5^	0.077	A	G	0.02	0.69	0.17	DEPTOR	0 (0)	0 (0)	0.16 (5)	0.01 (1)
13:18913685	3.42 × 10^−5^	0.077	A	G	0.02	0.69	0.17	DEPTOR	0 (0)	0 (0)	0.16 (5)	0.01 (1)
13:20734477	7.72 × 10^−5^	0.087	A	G	0.03	0.53	0.13	LOC111098540	0 (0)	0 (0)	0.23 (7)	0.03 (3)
14:3945297	5.97 × 10^−5^	0.084	A	G	0.07	0.36	0.09	EXOC4	0.1 (3)	0 (0)	0.19 (6)	0.08 (10)
14:4095927	5.97 × 10^−5^	0.084	A	G	0.07	0.36	0.09	-	0.1 (3)	0 (0)	0.19 (6)	0.08 (10)
14:54260890	8.46 × 10^−5^	0.084	A	G	0.02	0.72	0.18	-	0 (0)	0 (0)	0.13 (4)	0.01 (1)
18:37737740	3.07 × 10^−4^	0.066	C	A	0.12	0.25	0.07	-	0.06 (2)	0 (0)	0.45 (14)	0.16 (19)
18:37862012	4.81 × 10^−4^	0.074	A	T	0.17	0.21	0.06	-	0.19 (6)	0 (0)	0.32 (10)	0.24 (28)
18:37867871	4.81 × 10^−4^	0.074	A	G	0.17	0.21	0.06	OR5B21	0.19 (6)	0 (0)	0.32 (10)	0.24 (28)
18:38233567	1.74 × 10^−4^	0.053	A	G	0.11	0.29	0.08	-	0.06 (2)	0 (0)	0.42 (13)	0.13 (15)
18:38350947	3.76 × 10^−4^	0.066	A	G	0.11	0.27	0.08	-	0.06 (2)	0 (0)	0.42 (13)	0.13 (16)
18:38456518	3.76 × 10^−4^	0.066	A	G	0.11	0.27	0.08	CTNND1	0.06 (2)	0 (0)	0.42 (13)	0.13 (16)
18:38502268	2.16 × 10^−4^	0.053	A	G	0.12	0.26	0.07	ZDHHC5	0.1 (3)	0 (0)	0.39 (12)	0.16 (19)
18:38507461	2.16 × 10^−4^	0.053	A	G	0.12	0.26	0.07	ZDHHC5	0.1 (3)	0 (0)	0.39 (12)	0.16 (19)
18:38510335	2.16 × 10^−4^	0.053	A	G	0.12	0.26	0.07	ZDHHC5	0.1 (3)	0 (0)	0.39 (12)	0.16 (19)
18:38704682	4.49 × 10^−5^	0.053	A	G	0.32	0.20	0.05	-	0.26 (8)	0.08 (9)	0.55 (17)	0.37 (44)
18:38719709	1.45 × 10^−4^	0.053	G	A	0.30	0.18	0.05	-	0.23 (7)	0.08 (9)	0.55 (17)	0.34 (41)
18:39140112	8.21 × 10^−5^	0.053	G	A	0.26	0.22	0.06	LOC100688997	0.16 (5)	0.03 (4)	0.58 (18)	0.34 (41)
18:39727438	3.38 × 10^−4^	0.066	G	A	0.31	0.17	0.05	-	0.29 (9)	0.08 (9)	0.45 (14)	0.35 (42)
18:40281122	8.35 × 10^−5^	0.053	A	G	0.41	0.18	0.05	-	0.42 (13)	0.13 (16)	0.42 (13)	0.45 (53)
18:40286669	9.66 × 10^−5^	0.053	G	A	0.42	0.18	0.05	-	0.42 (13)	0.13 (16)	0.42 (13)	0.47 (56)
18:40418483	2.44 × 10^−4^	0.056	A	G	0.47	0.17	0.05	-	0.42 (13)	0.16 (19)	0.48 (15)	0.52 (62)
18:40653765	2.05 × 10^−4^	0.053	A	C	0.47	0.18	0.05	-	0.45 (14)	0.15 (18)	0.45 (14)	0.53 (63)
18:40667579	1.95 × 10^−4^	0.053	C	A	0.46	0.17	0.05	-	0.45 (14)	0.15 (18)	0.45 (14)	0.5 (60)
18:41229735	6.77 × 10^−5^	0.053	A	G	0.43	0.19	0.05	LOC100684610	0.45 (14)	0.13 (15)	0.42 (13)	0.49 (58)
18:41436427	1.54 × 10^−4^	0.053	C	A	0.52	−0.17	0.05	-	0.1 (3)	0.35 (42)	0.42 (13)	0.45 (53)
18:41713196	4.34 × 10^−4^	0.073	A	G	0.15	0.24	0.07	-	0.06 (2)	0.03 (3)	0.45 (14)	0.18 (21)
18:41726488	1.92 × 10^−4^	0.053	A	G	0.15	0.26	0.07	-	0.06 (2)	0.02 (2)	0.45 (14)	0.18 (22)
18:41854962	1.82 × 10^−4^	0.053	C	G	0.13	0.24	0.06	-	0.1 (3)	0.01 (1)	0.32 (10)	0.17 (20)
18:42270324	3.78 × 10^−4^	0.066	A	G	0.07	0.33	0.09	-	0 (0)	0.01 (1)	0.35 (11)	0.08 (9)
27:3054141	8.03 × 10^−4^	0.096	A	G	0.22	0.18	0.05	-	0.13 (4)	0.04 (5)	0.55 (17)	0.25 (30)
27:3154712	2.20 × 10^−4^	0.041	A	G	0.34	0.18	0.05	FIGNL2	0.26 (8)	0.08 (10)	0.55 (17)	0.4 (48)
27:3197949	7.78 × 10^−5^	0.027	G	A	0.18	0.23	0.06	SCN8A	0.13 (4)	0.03 (3)	0.48 (15)	0.21 (25)
27:3234647	4.84 × 10^−5^	0.027	G	A	0.17	0.24	0.06	SCN8A	0.13 (4)	0.03 (3)	0.48 (15)	0.19 (23)
27:3501246	1.13 × 10^−4^	0.027	C	A	0.38	−0.18	0.05	-	0.03 (1)	0.2 (24)	0.29 (9)	0.47 (56)
27:3594560	2.29 × 10^−5^	0.027	G	A	0.14	0.27	0.06	BIN2	0.13 (4)	0 (0)	0.45 (14)	0.18 (21)
27:4561667	7.57 × 10^−4^	0.095	A	C	0.23	0.18	0.05	LIMA1	0.13 (4)	0.05 (6)	0.58 (18)	0.25 (30)
27:5112245	6.08 × 10^−5^	0.027	A	G	0.22	0.20	0.05	SPATS2	0.29 (9)	0.02 (2)	0.39 (12)	0.27 (32)
27:5364442	4.76 × 10^−5^	0.027	A	G	0.17	0.24	0.06	LOC111092735	0.16 (5)	0.01 (1)	0.42 (13)	0.22 (26)
27:5467028	1.07 × 10^−4^	0.027	G	A	0.36	0.18	0.05	-	0.32 (10)	0.09 (11)	0.52 (16)	0.41 (49)
27:5478927	2.36 × 10^−4^	0.042	A	G	0.30	0.19	0.05	LMBR1L	0.23 (7)	0.03 (4)	0.52 (16)	0.43 (51)
27:5512765	5.03 × 10^−4^	0.072	A	G	0.36	0.17	0.05	RHEBL1	0.29 (9)	0.08 (9)	0.52 (16)	0.47 (56)
27:5603116	4.20 × 10^−4^	0.063	A	G	0.37	0.17	0.05	WNT10B	0.42 (13)	0.05 (6)	0.35 (11)	0.52 (62)
27:5806033	1.17 × 10^−4^	0.027	A	G	0.19	0.24	0.06	CCNT1	0.13 (4)	0.01 (1)	0.55 (17)	0.25 (30)
27:5817551	1.17 × 10^−4^	0.027	G	A	0.19	0.24	0.06	-	0.13 (4)	0.01 (1)	0.55 (17)	0.25 (30)
27:5822515	1.17 × 10^−4^	0.027	G	A	0.19	0.24	0.06	-	0.13 (4)	0.01 (1)	0.55 (17)	0.25 (30)
27:7188905	5.73 × 10^−4^	0.078	A	G	0.27	0.18	0.05	-	0.23 (7)	0.04 (5)	0.48 (15)	0.34 (41)
27:8331252	3.49 × 10^−5^	0.027	G	A	0.11	0.30	0.07	-	0.06 (2)	0 (0)	0.45 (14)	0.13 (15)
27:8646723	8.78 × 10^−5^	0.027	G	A	0.13	0.28	0.07	-	0.06 (2)	0 (0)	0.45 (14)	0.17 (20)
27:8767784	1.51 × 10^−4^	0.030	T	A	0.13	0.26	0.07	LOC111092890	0.06 (2)	0 (0)	0.45 (14)	0.18 (21)
27:8842830	2.80 × 10^−4^	0.044	A	C	0.12	0.24	0.07	LOC111092890	0.06 (2)	0.01 (1)	0.45 (14)	0.14 (17)
27:8883501	1.39 × 10^−4^	0.030	A	G	0.14	0.24	0.06	ANO6	0.13 (4)	0.01 (1)	0.42 (13)	0.17 (20)
27:8884575	2.80 × 10^−4^	0.044	A	G	0.12	0.24	0.07	ANO6	0.06 (2)	0.01 (1)	0.45 (14)	0.14 (17)
27:8892980	6.60 × 10^−5^	0.027	C	G	0.22	0.21	0.05	ANO6	0.16 (5)	0.04 (5)	0.55 (17)	0.25 (30)
27:11672173	6.00 × 10^−4^	0.078	A	C	0.16	0.23	0.07	LOC106557903	0.03 (1)	0 (0)	0.55 (17)	0.24 (28)

**Table 6:** Frequency of homozygosity in lymphoma cases (N = 31) and controls (N = 119) at highly significant haplotypes (*p* < 0.0001) from a logistic regression of haplotype blocks for chromosome 18. Genes in the region were fetched from NCBI (CanFam3.1, assembly GCF_000002285.3). Lym = lymphoma; Cont = controls.

No. of SNPs	Start SNP	End SNP	Haplotype	Odds Ratio	*p*-Value	Genes in Region	Frequency of Homozygosity (No. of Dogs)	
							Lym.	Cont.
14	18:38233567	18:38383741	AAGAGGGAAAAAGG	5.61	7.66 × 10^−5^	LOC483485, LOC610080, LOC106559985, OR10C10, LOC610127, COR5BA2	0.06 (2)	0 (0)
3	18:38502268	18:38510335	AAA	4.91	7.86 × 10^−5^	ZDHHC5	0.1 (3)	0 (0)
3	18:38502268	18:38510335	GGG	0.204	7.86 × 10^−5^	ZDHHC5	0.52 (16)	0.84 (100)
2	18:37862012	18:37867871	AA	3.98	9.97 × 10^−5^	OR5B21	0.19 (6)	0 (0)
2	18:37862012	18:37867871	TG	0.251	9.97 × 10^−5^	OR5B21	0.48 (15)	0.76 (91)

**Table 7:** Frequency of homozygosity in lymphoma cases (N = 31) and controls (N = 119) at highly significant haplotypes (*p* < 0.0001) from a logistic regression of haplotype blocks for chromosome 27. Genes in the region were fetched from the NCBI (CanFam3.1, assembly GCF_000002285.3). Lym = lymphoma; Cont = controls.

No. of SNPs	Start SNP	End SNP	Haplotype	Odds Ratio	*p*-Value	Genes in Region	Frequency of Homozygosity (No. of Dogs)	
							Lym.	Cont.
8	27:5112245	27:5241391	AAAGCGAA	4.94	2.82 × 10^−6^	SPATS2	0.29 (9)	0.02 (2)
2	27:3590788	27:3594560	AG	6.26	5.33 × 10^−6^	BIN2	0.13 (4)	0 (0)
12	27:3211312	27:3347721	TCGAGGAGGGGC	5.95	7.53 × 10^−6^	SCN8A	0.13 (4)	0 (0)
2	27:5806033	27:5817551	AG	5.31	1.51 × 10^−5^	CCNT1	0.13 (4)	0.01 (1)
2	27:5806033	27:5817551	GA	0.188	1.51 × 10^−5^	CCNT1	0.32 (10)	0.74 (88)
4	27:8752431	27:8792430	GTGG	5.24	6.12 × 10^−5^	LOC111092890	0.06 (2)	0 (0)
2	27:5592820	27:5603116	CA	3.65	6.83 × 10^−5^	WNT10B	0.42 (13)	0.05 (6)

**Table 8:** REML results for the proportion of phenotypic variance explained by all autosomal SNPs, significant chromosomes, and regions within each significant chromosome. V(G)/Vp indicates proportion of phenotypic variance (Vp) over genetic variance (V(G)) (heritability). V(G)/Vp_L is heritability transformed to the underlying liability scale. SE: standard error.

Chromosome	Prevalence	V(G)/Vp ± SE (%)	V(G)/Vp_L ± SE (%)	*p*-Value
All autosomes	0.025	100 ± 20.52	106.09 ± 21.77	1.43 × 10^−6^
	0.05		129.37 ± 26.55	
	0.1		160.4 ± 32.92	
Chr 18	0.025	31.27 ± 12.77	33.17 ± 13.54	1.21 × 10^−3^
	0.05		40.45 ± 16.52	
	0.1		50.16 ± 20.48	
Chr 18 37 to 56 Mb region	0.025	40.91 ± 12.1	43.4 ± 12.83	7.10 × 10^−6^
	0.05		52.93 ± 15.65	
	0.1		65.63 ± 19.4	
Chr 27	0.025	40.71 ± 11.26	43.19 ± 11.94	1.34 × 10^−6^
	0.05		52.67 ± 14.56	
	0.1		65.3 ± 18.06	
Chr 27 1 to 9 Mb region	0.025	26.12 ± 9.6	27.71 ± 10.18	2.50 × 10^−7^
	0.05		33.79 ± 12.42	
	0.1		41.9 ± 15.4	

## Data Availability

The data presented in this study are available in the supplementary files (Supplementary Data S3 and S4).

## Supplementary Materials

The following supporting information can be downloaded at https://www.mdpi.com/article/10.3390/vetsci10090581/s1, Supplementary Data S1, sample information; Supplementary Data S2, REML results; Supplementary Data S3, PLINK genotype files (.bed, .bim, .fam) for dogs genotyped on the 170 k array; Supplementary Data S4, PLINK genotype files (.bed, .bim, .fam) for dogs genotyped on the 220 k array; Supplementary Figures S1–S3, regional association plots for chromosome 27; Supplementary Table S1, linear discriminant analysis results for the top 100 SNPs using a quantitative phenotype; Supplementary Table S2, linear discriminant analysis results for the top 100 SNPs using a binary phenotype.
