# Supplementary material for: Lymphoma in Border Collies: Genome-Wide Association and Pedigree Analysis
Source: Vet Sci. 2023 Sep 19;10(9):581. doi: 10.3390/vetsci10090581 (PMC10536503; doi:10.3390/vetsci10090581)
Supplement: Supplementary file 1 [file vetsci-10-00581-s001.zip › vetsci-2555740-supplementary.pdf]

veterinary sciences

MDPI

SUPPLEMENTARY DATA

Supplementary Data S1. Details for (A) genotyped dogs, and (B) lymphoma-affected dogs

(A) Genotyped dogs

DogID	Genotype ID	Lymphoma	Sex	Sire	Dam	Year of birth
dog_45527	B1		F	dog_45405	dog_45433	2003
dog_63658	B10		M	dog_50712	dog_28335	2008
dog_70670	B11		F	dog_56199	dog_8197	1999
dog_63659	B12		F	dog_50712	dog_28335	2008
dog_51018	B13		F	dog_50743	dog_20043	2008
dog_62810	B3		F	dog_51768	dog_16498	2001
dog_51015	B15		F	dog_50743	dog_20043	2008
dog_64294	B16		F	dog_52123	dog_46413	NA
dog_66825	B17			dog_11388	dog_5121	2003
dog_71936	B18		F	dog_2929	dog_71929	
dog_65970	B19		F	dog_43742	dog_51271	2008
dog_45472	B2		M	dog_16360	dog_59900	1997
dog_65971	B20		M	dog_43742	dog_51271	2008
dog_21402	B21		F	dog_26336	dog_21106	2006
dog_67774	B22			dog_67767	dog_68029	NA
dog_60438	B23		F	dog_8279	dog_48974	2007
dog_33573	B24		F	dog_16128	dog_56091	2002
dog_35051	B25		F	dog_76305	dog_33124	2007
dog_32328	B26		M	dog_37744	dog_26960	2008
dog_59021	B35		M	dog_83464	dog_58773	2010
dog_20940	B28					2009
dog_64264	B29		M	dog_64506	dog_63118	2005
dog_45502	PBC11		F	dog_1728	dog_45435	2002
dog_14762	PBC01		F	dog_1352	dog_1373	1995
dog_3819	B31	No	M	dog_2535	dog_2554	2009
dog_40138	B32		M	dog_40324	dog_81186	2009
dog_40470	B33		M	dog_40324	dog_81186	2009
dog_57834	B34		M	dog_37301	dog_57832	2007
dog_78557	B36		M	dog_4189	dog_76250	2010
dog_70671	B43		F	dog_56199	dog_8197	1999
dog_38609	B38		F	dog_16437	dog_38608	2004
dog_15401	B39		M	dog_2540	dog_4019	2001
dog_74189	B4		M	dog_39166	dog_31890	2002
dog_15397	B40		F	dog_81117	dog_70669	2001
dog_70669	B41		F	dog_56199	dog_52097	1998

DogID	Genotype ID	Lymphoma	Sex	Sire	Dam	Year of birth
dog_80869	B42		M	dog_38061	dog_80868	2003
dog_37595	B44		F	dog_41530	dog_7859	2006
dog_39525	B5		F	dog_2451	dog_52663	
dog_56199	B6		M	dog_1964	dog_56538	1995
dog_63657	B7		M	dog_50712	dog_28335	2008
dog_63656	B8		F	dog_50712	dog_28335	2008
dog_39216	B9		M	dog_24350	dog_24351	1997
dog_2208	BC1	Yes	F	dog_2202	dog_2207	2010
dog_58840	BC10		F	dog_1368	dog_58890	2013
dog_58783	BC11		F	dog_58886	dog_58784	2012
dog_12390	BC12		M	dog_82666	dog_40569	2014
dog_26887	BC13		F	dog_3662	dog_69176	2012
dog_27639	BC14		M	dog_3819	dog_27646	2013
dog_21687	BC15		M	dog_7964	dog_61146	2009
dog_6186	BC16			dog_3360	dog_43465	2010
dog_29765	BC17		M			
dog_34459	BC18		M			2012
dog_52215	BC19		F	dog_47453	dog_54734	2014
dog_28663	BC2	Yes	F	dog_14936	dog_28655	2008
dog_8231	BC3		F	dog_42999	dog_32054	
dog_58820	BC4		F	dog_58917	dog_58772	2012
dog_61258	BC5		M	dog_78943	dog_55630	2009
dog_31594	BC6		M	dog_2459	dog_68029	2010
dog_58756	BC7		M	dog_58886	dog_58812	2013
dog_59020	BC8		M	dog_83464	dog_58773	2010
dog_27550	BC9		M	dog_3820	dog_3821	2012
dog_2054	PBC02		F	dog_950	dog_2053	1997
dog_2781	PBC07		M	dog_1350	dog_2779	1996
dog_27710	PBC03		F	dog_2555	dog_27736	2012
dog_58956	PBC04		F			
dog_51235	PBC216	Yes	M			2017
dog_8758	PBC06	Yes	M			2003
dog_27936	PBC107		F	dog_3739	dog_35435	2000
dog_64553	PBC08		M	dog_64653	dog_81269	2004
dog_10584	PBC09		F	dog_42539	dog_24580	2010
dog_67215	PBC097	Yes	M			2004
dog_2624	PBC100	Yes	F	dog_2502	dog_2623	2013
dog_2619	PBC099	Yes	M	dog_2556	dog_2566	2008
dog_54769	PBC10		F	dog_75960	dog_3890	2013
dog_53836	PBC098	Yes	F			2009

DogID	Genotype ID	Lymphoma	Sex	Sire	Dam	Year of birth
dog_2629	PBC162	Yes	F	dog_2599	dog_2628	2009
dog_2634	PBC157	Yes	M	dog_2573	dog_2617	2009
dog_2373	PBC103	Yes	M	dog_2309	dog_2372	2002
dog_2557	PBC104	Yes	F	dog_2463	dog_2466	1997
dog_2199	PBC109		M	dog_2195	dog_2092	2002
dog_2555	PBC110		M	dog_2453	dog_2548	2004
dog_2311	PBC169		F	dog_1381	dog_2055	1993
dog_2521	PBC177		M	dog_2463	dog_2520	1998
dog_951	PBC122		M	dog_947	dog_945	1992
dog_1986	PBC184		M	dog_1965	dog_953	1997
dog_1365	PBC180		F	dog_80951	dog_1329	1991
dog_2315	PBC125		M	dog_2313	dog_952	1999
dog_51019	B14		M	dog_50743	dog_20043	2008
dog_74020	PBC108		F	dog_14936	dog_74024	2012
dog_3761	PBC114		M	dog_2787	dog_2372	2000
dog_75982	PBC115		M	dog_3761	dog_3926	2011
dog_3821	PBC152	No	F	dog_2780	dog_2612	2005
dog_32918	PBC16		F	dog_1351	dog_37661	2001
dog_18068	PBC118		F	dog_14546	dog_2746	2004
dog_67880	PBC32		F	dog_44458	dog_2279	2011
dog_64578	PBC12		M	dog_64626	dog_81216	2004
dog_72407	PBC91		M	dog_2631	dog_14913	2006
dog_24990	PBC135		F	dog_2579	dog_3791	2009
dog_2782	PBC112		F	dog_2781	dog_1374	2000
dog_27575	PBC113		F	dog_2315	dog_1374	2003
dog_17516	PBC117		F	dog_2373	dog_18068	2011
dog_81266	PBC120		M	dog_3761	dog_2585	2008
dog_1919	PBC123		M	dog_1913	dog_1912	1991
dog_2174	PBC124		F	dog_1913	dog_1924	1991
dog_59266	PBC134		F	dog_2635	dog_30395	2009
dog_27574	PBC138		F	dog_3800	dog_1374	2005
dog_44704	PBC13		F	dog_3705	dog_27649	2012
dog_3820	PBC139	No	M	dog_3800	dog_1374	2005
dog_48468	PBC166		F	dog_3882	dog_2629	2011
dog_79349	PBC132		F	dog_3808	dog_31573	2011
dog_3706	PBC133	No	F	dog_3705	dog_2783	2009
dog_28655	PBC105		F	dog_2781	dog_27936	2004
dog_14936	PBC106		M	dog_2854	dog_14762	2004
dog_2384	PBC126	No	M	dog_2376	dog_2383	1990
dog_28656	PBC137		M	dog_2459	dog_27936	2006

DogID	Genotype ID	Lymphoma	Sex	Sire	Dam	Year of birth
dog_2586	PBC130		F	dog_1986	dog_2052	2003
dog_2583	PBC172		F	dog_1444	dog_2582	2001
dog_44746	PBC14		M	dog_3705	dog_27649	2012
dog_28043	PBC140		F	dog_37667	dog_37626	2011
dog_3818	PBC141	No	F	dog_3662	dog_3789	2012
dog_7770	PBC142		M	dog_2929	dog_82702	2005
dog_3756	PBC143	No	F	dog_3755	dog_3277	2011
dog_40477	PBC144		F	dog_30590	dog_40491	2012
dog_83943	PBC145		F	dog_82701	dog_69189	2012
dog_68158	PBC146		M	dog_3119	dog_71460	2008
dog_24661	PBC147		F	dog_3586	dog_24652	2010
dog_53847	PBC148		F	dog_21483	dog_53846	2011
dog_70570	PBC229		M	dog_62813	dog_70562	2012
dog_46131	PBC15		M	dog_44746	dog_46106	2014
dog_72408	PBC150		F	dog_27662	dog_72411	2013
dog_2540	PBC173		M	dog_1351	dog_1365	1998
dog_1368	PBC111		M	dog_911	dog_1365	1993
dog_67662	PBC153		M	dog_67975	dog_68069	2011
dog_13545	PBC154		M	dog_25056	dog_11665	2009
dog_71131	PBC155		F	dog_10017	dog_56764	2011
dog_52791	PBC156		F	dog_36661	dog_6068	2010
dog_68422	PBC163	Yes	F			2008
dog_2609	PBC158	Yes	M	dog_2308	dog_2608	2007
dog_2618	PBC159	Yes	F	dog_1660	dog_2156	2005
dog_2150	PBC168		M	dog_2139	dog_2149	2000
dog_27418	PBC160	Yes	F			2012
dog_58091	PBC161	Yes	M			2008
dog_38666	PBC218	Yes	M			2010
dog_2446	PBC05	Yes	F	dog_2430	dog_2444	2005
dog_2622	PBC164	Yes	F	dog_2458	dog_2621	2002
dog_5456	PBC165	Yes	F			2004
dog_2308	PBC170		M	dog_2307	dog_2306	1998
dog_3882	PBC167	No	M	dog_3586	dog_3878	2007
dog_2608	PBC171		F	dog_2504	dog_2482	2005
dog_1658	PBC178		M	dog_1483	dog_1580	1994
dog_13183	PBC17		F	dog_13214	dog_13179	2011
dog_2430	PBC129		M	dog_2141	dog_2424	2000
dog_79429	PBC121		F	dog_3808	dog_31573	2011
dog_48833	PBC136		F	dog_24378	dog_2509	2013
dog_3234	PBC116		M	dog_2309	dog_2372	2002

DogID	Genotype ID	Lymphoma	Sex	Sire	Dam	Year of birth
dog_10708	PBC174		F	dog_55234	dog_8145	2013
dog_28657	PBC175		F	dog_2459	dog_27936	2006
dog_27553	PBC176		F	dog_3796	dog_3821	2009
dog_43714	PBC119		M	dog_2463	dog_2466	1997
dog_45763	PBC127		F	dog_1973	dog_1722	2001
dog_68625	PBC179		F	dog_2599	dog_2628	2009
dog_81434	PBC18		F	dog_81476	dog_81515	2009
dog_10852	PBC131		F	dog_2530	dog_2559	2002
dog_2638	PBC181	Yes	M	dog_2579	dog_2637	2008
dog_9938	PBC182	Yes	M			2006
dog_47270	PBC183	Yes	M			2008
dog_3420	PBC128		F	dog_2430	dog_2444	2004
dog_3063	PBC185	No	M	dog_3061	dog_3060	1992
dog_3770	PBC186		M	dog_3739	dog_3023	1995
dog_3072	PBC187	No	M	dog_1730	dog_2167	1989
dog_83455	PBC188	Yes	F	dog_70570	dog_83443	2014
dog_42023	PBC189	Yes	F			2007
dog_67514	PBC19		M	dog_67506	dog_62026	2004
dog_78248	PBC190	Yes	F	dog_58640	dog_40701	2011
dog_25130	PBC191	Yes	M			2008
dog_4429	PBC192	Yes	F	dog_4431	dog_4537	2010
dog_33205	PBC193	Yes	M			2011
dog_26886	PBC194		F	dog_3662	dog_69176	2014
dog_28042	PBC195		M	dog_76668	dog_26887	2015
dog_28666	PBC196		F	dog_24524	dog_28657	2011
dog_28670	PBC197		F	dog_45782	dog_28666	2015
dog_26656	PBC198					2012
dog_60480	PBC199		M			2015
dog_81513	PBC20		F	dog_81547	dog_81515	2010
dog_27540	PBC200		M	dog_3820	dog_3706	2014
dog_27546	PBC201		F	dog_72593	dog_27602	2015
dog_59945	PBC202		M	dog_63204	dog_11122	2016
dog_59949	PBC203		F	dog_63204	dog_11122	2016
dog_59948	PBC204		M	dog_11121	dog_11122	2014
dog_11122	PBC205		F	dog_60141	dog_45891	2011
dog_11121	PBC206		M	dog_8789	dog_33345	2009
dog_59947	PBC207		F	dog_11121	dog_11122	2014
dog_59944	PBC208		F	dog_20979	dog_11122	2013
dog_59946	PBC209		M	dog_20979	dog_11122	2013
dog_12065	PBC21		M	dog_78366	dog_12066	2012

DogID	Genotype ID	Lymphoma	Sex	Sire	Dam	Year of birth
dog_33172	PBC210		M			2011
dog_69898	PBC211		F	dog_69932	dog_79388	2016
dog_72411	PBC212		F	dog_2631	dog_14913	2006
dog_2783	PBC213		F	dog_1377	dog_2782	2003
dog_13339	PBC214	Yes	F	dog_18222	dog_13422	2008
dog_13422	PBC215	Yes	F	dog_2361	dog_3678	2006
dog_1370	PBC101	Yes	F	dog_1368	dog_1367	1995
dog_6612	PBC217	Yes	F	dog_2150	dog_6307	2007
dog_1374	PBC102	Yes	F	dog_1351	dog_1370	1997
dog_84063	PBC228	Yes	M			2007
dog_39723	PBC22		F	dog_57664	dog_39692	2008
dog_6307	PBC220		F	dog_2141	dog_6572	2000
dog_29008	PBC221		M	dog_28953	dog_28925	
dog_24693	PBC222		M	dog_54836	dog_12404	1997
dog_74713	PBC223		F	dog_29034	dog_33386	
dog_59937	PBC224		F	dog_54836	dog_59930	1998
dog_6754	PBC225		M	dog_3381	dog_3353	2002
dog_37960	PBC226		M	dog_16360	dog_2257	1994
dog_84004	PBC227	Yes	M	dog_4907	dog_59711	2008
dog_23911	PBC219	Yes	M			2006
dog_82888	B30		M	dog_36129	dog_82880	2011
dog_67122	PBC23		F	dog_3628	dog_69064	2012
dog_23346	PBC230		F	dog_50125	dog_23349	2004
dog_8893	PBC24		F	dog_82020	dog_40438	2014
dog_3627	PBC25		M	dog_858	dog_3346	1996
dog_77493	PBC26		F	dog_2313	dog_77551	2007
dog_4586	PBC27		M	dog_76827	dog_39694	2009
dog_78403	PBC28					2014
dog_78402	PBC29					2011
dog_71922	PBC30		M	dog_3866	dog_71936	2011
dog_54082	PBC149		F	dog_54084	dog_82970	2006
dog_31133	PBC151		M	dog_60464	dog_60462	2011
dog_31606	PBC31		M	dog_19177	dog_5985	2013
dog_57963	PBC34		M			2012
dog_74385	PBC35		F			2013
dog_60437	PBC33		F	dog_60436	dog_60439	2012
dog_67148	PBC36		M	dog_79713	dog_60459	2014
dog_70164	PBC38		F			1995
dog_50771	PBC39		M			1995
dog_77145	PBC40		F	dog_1872	dog_77045	2010

DogID	Genotype ID	Lymphoma	Sex	Sire	Dam	Year of birth
dog_60463	PBC37		F	dog_78362	dog_29404	2010
dog_30736	PBC41		M	dog_36129	dog_30738	2011
dog_64024	PBC42		F	dog_64025	dog_60454	2011
dog_60455	PBC43		F	dog_47188	dog_36117	2011
dog_76110	PBC44		M	dog_18696	dog_76109	2008
dog_31574	PBC46		M	dog_2459	dog_12507	2005
dog_54776	PBC47		F	dog_2535	dog_54737	2013
dog_45350	PBC48		M	dog_45304	dog_45267	2010
dog_58187	PBC45		F	dog_48910	dog_26634	2008
dog_58782	PBC50		M	dog_2985	dog_1918	2003
dog_66979	PBC51		F	dog_3540	dog_80834	2003
dog_28632	PBC52		F	dog_28602	dog_28610	2002
dog_66434	PBC53		M	dog_18187	dog_3541	2008
dog_59006	PBC54		M	dog_58971	dog_62824	2009
dog_62824	PBC55		F	dog_1921	dog_62819	2005
dog_40422	PBC56		F	dog_2277	dog_40431	2009
dog_58917	PBC57		M	dog_62828	dog_62824	2006
dog_60310	PBC58		M	dog_57220	dog_60309	2011
dog_6196	PBC59		F	dog_3360	dog_3409	2009
dog_58379	PBC60		F	dog_44519	dog_42664	2012
dog_73865	PBC61		M	dog_64996	dog_46792	2011
dog_28479	PBC62		F	dog_3545	dog_3456	2012
dog_10507	PBC63		F	dog_58878	dog_11545	2012
dog_66867	PBC64		M	dog_66929	dog_66861	2011
dog_6499	PBC65		F	dog_4222	dog_6870	2011
dog_47849	PBC66		F	dog_47868	dog_47858	2010
dog_69171	PBC67		M	dog_55234	dog_69191	2011
dog_54805	PBC68		M	dog_75960	dog_3890	2013
dog_6650	PBC69		M	dog_40441	dog_6611	2012
dog_58888	PBC70		F	dog_58886	dog_17480	2012
dog_79388	PBC71		F	dog_3808	dog_31573	2013
dog_42641	PBC72		M	dog_3603	dog_42661	2012
dog_61248	PBC73		M	dog_51210	dog_13505	2011
dog_40569	PBC74		F	dog_77147	dog_77336	2012
dog_73855	PBC75		M	dog_73861	dog_73863	2014
dog_3816	PBC76		M	dog_3721	dog_3717	2013
dog_45782	PBC77		M	dog_28055	dog_45785	2012
dog_79949	PBC78		M	dog_20956	dog_31603	2014
dog_79927	PBC79		M	dog_20956	dog_31603	2014
dog_26963	PBC80		M	dog_24554	dog_50928	2011

DogID	Genotype ID	Lymphoma	Sex	Sire	Dam	Year of birth
dog_44947	PBC81		F	dog_3868	dog_44918	2010
dog_44946	PBC82		M	dog_41765	dog_3451	2010
dog_9699	PBC49		F	dog_10017	dog_56764	2011
dog_3823	PBC84	No	M	dog_3820	dog_3706	2014
dog_83021	PBC85		M	dog_3705	dog_27724	2016
dog_83023	PBC86		F	dog_3705	dog_27724	2016
dog_83019	PBC87		F	dog_3705	dog_27724	2016
dog_83022	PBC88		F	dog_3705	dog_27724	2016
dog_32793	PBC89		M	dog_62550	dog_38685	2013
dog_72410	PBC90		M	dog_3705	dog_72411	2011
dog_55778	PBC83		F	dog_9700	dog_79073	2013
dog_14972	PBC92		M	dog_14947	dog_27515	2013
dog_75629	PBC93		F	dog_75628	dog_75608	2012
dog_77263	PBC94		F	dog_63218	dog_77002	2012
dog_77336	PBC95		F	dog_2309	dog_77144	2006
dog_79512	PBC96		M	dog_59274	dog_79429	2014

Supplementary Data S1 (continued)

(B) Lymphoma-affected dogs

**Dog ID**	**Genotype ID**	**Sex**	**Sire**	**Dam**	**Year of birth**	**Age diagnosed**	**Lymphoma type**	**Stage**	**Grade**	**Localisation**	**Treatment**	**Treatment outcome**	**Age of death**
dog_2208	BC1	F	dog_2202	dog_2207	2010	3y6m	B-cell	I	Low	Multicentric	Chemotherapy	Recurrence	
dog_4429	PBC192	F	dog_4431	dog_4537	2010	8y2m	Unknown type				Chemotherapy	Complete remission	
dog_2286		F	dog_1512	dog_2285	2001	12y10m	Unknown type			Multicentric	Palliative		13y2m
dog_6612	PBC217	F	dog_2150	dog_6307	2007	12y	B-cell				Chemotherapy	Still undergoing treatment	
dog_8758	PBC06	M			2003	12y8m	T-cell	V	High	Cutaneous	Chemotherapy	Partial remission	12y11m
dog_1751		F	dog_904	dog_1745	1984	11y	Unknown type	V		Alimentary	No treatment		11y
dog_9672		M			1900	5y	Unknown type		High	Multicentric	Unknown		6y
dog_9938	PBC182	M			2006	10y11m	B-cell	III	High	Multicentric	Chemotherapy	Partial remission	
dog_2558		M	dog_2313	dog_2392	1998	9y10m	Unknown type			Alimentary	Unknown		10y
dog_2560		F	dog_2530	dog_2559	2002	11y2m	B-cell	I	Low	Multicentric	Chemotherapy		11y6m
dog_13339	PBC214	F	dog_18222	dog_13422	2008	8y1m	T-cell			Cutaneous	Chemotherapy	Replased, still undergoing treatment	
dog_13422	PBC215	F	dog_2361	dog_3678	2006	9y6m	Unknown type			Multicentric	Chemotherapy	Complete remission	
dog_13511		M	dog_2361	dog_3678	2006	7y10m	Unknown type			Cutaneous	Chemotherapy then palliative		8y
dog_84004	PBC227	M	dog_4907	dog_59711	2008	11y	B-cell			Multicentric	Unknown		
dog_2604		M	dog_1920	dog_2603	2002	12y3m	B-cell	III	High	Multicentric	Chemotherapy		12y7m
dog_1913		M	dog_928	dog_1908	1985	12y10m	Unknown type				Palliative		13y
dog_22693		F			1998	12y6m	Unknown type	V	High	Multicentric	Palliative		12y7m
dog_23911	PBC219	M			2006	13y4m	Unknown type				Palliative		

**Dog ID**	**Genotype ID**	**Sex**	**Sire**	**Dam**	**Year of birth**	**Age diagnosed**	**Lymphoma type**	**Stage**	**Grade**	**Localisation**	**Treatment**	**Treatment outcome**	**Age of death**
dog_27418	PBC160	F			2012	5y1m	B-cell		High	Multicentric	Chemotherapy	Still undergoing treatment	
dog_1374	PBC102	F	dog_1351	dog_1370	1997	13y	Unknown type	I	Low		Palliative		14y
dog_28663	BC2	F	dog_14936	dog_28655	2008	6y	Unknown type				Unknown		6y
dog_30475		F	dog_33176	dog_22634	2011	7y10m	Unknown type				Unknown		7y10m
dog_2614		M	dog_2540	dog_2613	2008	5y	Unknown type		High	Multicentric	Chemotherapy		6y
dog_31795		M	dog_30018	dog_4188	2006	10y	Unknown type				Unknown		10y9m
dog_33176		M	dog_50125	dog_24226	2007	4y11m	Unknown type				Unknown		5y
dog_33205	PBC193	M			2011	7y3m	Unknown type				Chemotherapy	Complete remission	
dog_38666	PBC218	M			2010	9y1m	B-cell		High		Chemotherapy	Still undergoing treatment	
dog_2587		F	dog_1658	dog_2586	2006	8y	B-cell	III	Intermediate	Multicentric	Chemotherapy		9y4m
dog_2468		F	dog_1788	dog_2467	1993	4y6m	B-cell	III		Multicentric	Chemotherapy		6.5y
dog_40443		F	dog_17538	dog_50122	2010	7y9m	Unknown type				Unknown		7y9m
dog_2609	PBC158	M	dog_2308	dog_2608	2007	11y5m	B-cell	III	High	Multicentric	Chemotherapy	Complete remission	
dog_2618	PBC159	F	dog_1660	dog_2156	2005	12y	T-cell	V		Cutaneous	Unknown		
dog_42023	PBC189	F			2007	11y	Unknown type	IVb		Multicentric	Palliative		
dog_2557	PBC104	F	dog_2463	dog_2466	1997	9y6m	Unknown type	II		Multicentric	Chemotherapy	Euthanased	9y6m
dog_2446	PBC05	F	dog_2430	dog_2444	2005	11y	B-cell	III	High	Multicentric	Chemotherapy	Complete remission	
dog_2445		F	dog_2430	dog_2444	2007	7y	Unknown type				Unknown		8y
dog_2619	PBC099	M	dog_2556	dog_2566	2008	9y	B-cell				Chemotherapy	Complete remission	
dog_2620		M	dog_1973	dog_1722	2001	14y7m	Chronic large cell lymphoma				No treatment		15y7m
dog_2622	PBC164	F	dog_2458	dog_2621	2002	15y3m	Unknown type				Unknown		
dog_2624	PBC100	F	dog_2502	dog_2623	2013	2y11m	B-cell	III		Multicentric	Chemotherapy	Complete remission	

Dog ID	Genotype ID	Sex	Sire	Dam	Year of birth	Age diagnosed	Lymphoma type	Stage	Grade	Localisation	Treatment	Treatment outcome	Age of death
dog_2626		F	dog_2082	dog_2625	2004	3y6m	Unknown type				Unknown		4y
dog_2625		F	dog_2574	dog_2616	2002	6y1m	Unknown type				Unknown		6y4m
dog_2615		F	dog_44286	dog_2399	2000	4y5m	Unknown type			Multicentric	No treatment		5y
dog_2630		F	dog_2354	dog_2515	1990	11y	Unknown type			Alimentary	Surgery		12y3m
dog_51235	PBC216	M			2017	1y11m	B-cell	V	Intermediate to high	Multicentric	Immunotherapy trial	Euthanased	1y11m
dog_2632		F	dog_2631	dog_2606	2002	13y6m	B-cell	IV		Multicentric	Unknown		13y8m
dog_2633		F	dog_1884	dog_2518	2007	9y11m	Unknown type				Unknown		9y11m
dog_53834		M	dog_50125	dog_19509	2005	4y	Unknown type				Unknown		5y
dog_53836	PBC098	F			2009	3y	B-cell				Unknown	Complete remission	
dog_55776		F			2001	9y	Unknown type	V			Surgery		10y
dog_2634	PBC157	M	dog_2573	dog_2617	2009	8y1m	B-cell			Multicentric	Palliative		8y2m
dog_58091	PBC161	M			2008	8y	B-cell			Multicentric	Chemotherapy	Still undergoing treatment	
dog_1370	PBC101	F	dog_1368	dog_1367	1995	12y	Unknown type	I	Low		Chemotherapy	Partial remission	13y
dog_2635		M	dog_2564	dog_2612	2009	6y4m	Unknown type		High		No treatment		6y4m
dog_2636		M	dog_2384	dog_2607	1994	10y	Unknown type	IV		Extranodal	Chemotherapy	Partial remission	12y
dog_59711		F	dog_50743	dog_64539	2005	11y	B-cell			Multicentric	Chemotherapy	Partial remission	12y6m
dog_2465		M	dog_1788	dog_2462	1994	13y	Unknown type			Multicentric	Palliative		13y2m
dog_67215	PBC097	M			2004	13y	Unknown type				Unknown	Unknown	
dog_2281		F	dog_2280	dog_2279	2006	10y9m	Unknown type			Multicentric	Palliative		
dog_68422	PBC163	F			2008	9y	Unknown type				Unknown	Euthanased	10y
dog_2629	PBC162	F	dog_2599	dog_2628	2009	4y	B-cell			Follicular	Unknown	Complete remission	
dog_2638	PBC181	M	dog_2579	dog_2637	2008	9y11m	B-cell	IV	High	Multicentric	Chemotherapy	Partial remission	
dog_2640		M	dog_2596	dog_2639	2003	6y11m	Unknown type	V	High	Multicentric	Unknown		7y

**Dog ID**	**Genotype ID**	**Sex**	**Sire**	**Dam**	**Year of birth**	**Age diagnosed**	**Lymphoma type**	**Stage**	**Grade**	**Localisation**	**Treatment**	**Treatment outcome**	**Age of death**
dog_84063	PBC228	M			2007	13y	B-cell				Unknown	Still undergoing treatment	
dog_2585		F	dog_2584	dog_2583	2004	9y5m	Unknown type			Cutaneous	Unknown		9y5m
dog_2641		F	dog_2579	dog_2541	2010	5y7m	B-cell		High	Multicentric	Unknown		5y8m
dog_76974		M	dog_41050		2001	10y	Unknown type	III	High	Cutaneous	No treatment		10y
dog_78248	PBC190	F	dog_58640	dog_40701	2011	6y8m	Unknown type	III		Multicentric	Unknown	Still undergoing treatment	
dog_2645		M	dog_2644	dog_2642	2011	5y	Unknown type				Unknown		6y
dog_2373	PBC103	M	dog_2309	dog_2372	2002	9y	B-cell	IV			Chemotherapy	Partial remission	9y8m
dog_83455	PBC188	F	dog_70570	dog_83443	2014	3y	Unknown type				Unknown		
dog_25130	PBC191	M			2008	7y11m	Acute lymphocytic leukaemia/Stage V lymphoma	V			Chemotherapy	Complete remission	
dog_5456	PBC165	F			2004	13y	Acute lymphocytic leukaemia/Stage V lymphoma	V	High		Unknown		
dog_47270	PBC183	M			2008	9y	T-cell, Acute lymphocytic leukaemia/Stage V lymphoma	Va			Unknown		

Supplementary Data S2. REML results for all chromosomes and regions of interest for a prevalence of (A) 0.1, (B) 0.05, and (C) 0.025.

(A) Prevalence 0.1

Chromosome/Region	V(G) Variance	V(G) SE	V(e) Variance	V(e) SE	Vp Variance	Vp SE	V(G)/Vp Variance	V(G)/Vp SE	V(G)/Vp_L Variance	V(G)/Vp_L SE	LogL	LogL0	LRT	df	Pval	n
1	0.0136	0.0193	0.1512	0.0249	0.1648	0.0192	0.0828	0.1161	0.1328	0.186	57.425	57.204	0.441	1	2.53E-01	150
2	0.0228	0.0208	0.1421	0.0237	0.1649	0.0194	0.1385	0.1219	0.2221	0.196	58.08	57.204	1.751	1	9.29E-02	150
3	0.0490	0.0260	0.1186	0.0223	0.1676	0.0209	0.2924	0.1380	0.4690	0.221	59.781	57.204	5.153	1	1.16E-02	150
4	0.0103	0.0157	0.1541	0.0233	0.1644	0.0191	0.0624	0.0952	0.1001	0.153	57.451	57.204	0.494	1	2.41E-01	150
5	0.0495	0.0251	0.1155	0.0224	0.1649	0.0202	0.3000	0.1364	0.4813	0.219	60.232	57.204	6.056	1	6.93E-03	150
6	0.0137	0.0166	0.1510	0.0228	0.1647	0.0192	0.0832	0.0993	0.1334	0.159	57.668	57.204	0.927	1	1.68E-01	150
7	0.0367	0.0235	0.1287	0.0232	0.1654	0.0199	0.2218	0.1326	0.3557	0.213	58.88	57.204	3.35	1	3.36E-02	150
8	0.0083	0.0136	0.1559	0.0223	0.1643	0.0190	0.0506	0.0822	0.0811	0.132	57.504	57.204	0.6	1	2.19E-01	150
9	0.0004	0.0121	0.1647	0.0229	0.1650	0.0191	0.0022	0.0733	0.0035	0.118	57.205	57.204	0.001	1	4.88E-01	150
10	0.0089	0.0135	0.1554	0.0219	0.1643	0.0191	0.0540	0.0814	0.0866	0.131	57.592	57.204	0.776	1	1.89E-01	150
11	0.0219	0.0188	0.1422	0.0228	0.1641	0.0192	0.1337	0.1110	0.2145	0.178	58.179	57.204	1.949	1	8.13E-02	150
12	0.0403	0.0218	0.1243	0.0207	0.1646	0.0201	0.2447	0.1196	0.3925	0.192	60.42	57.204	6.431	1	5.61E-03	150
13	0.0151	0.0158	0.1487	0.0222	0.1638	0.0191	0.0924	0.0949	0.1481	0.152	57.911	57.204	1.413	1	1.17E-01	150
14	0.0453	0.0227	0.1190	0.0200	0.1643	0.0205	0.2755	0.1217	0.4419	0.195	61.495	57.204	8.582	1	1.70E-03	150
15	0.0066	0.0123	0.1579	0.0218	0.1645	0.0191	0.0401	0.0746	0.0643	0.120	57.4	57.204	0.392	1	2.66E-01	150
16	0.0004	0.0112	0.1646	0.0224	0.1650	0.0191	0.0026	0.0678	0.0042	0.109	57.205	57.204	0.001	1	4.86E-01	150
17	0.0399	0.0210	0.1233	0.0204	0.1632	0.0198	0.2446	0.1166	0.3924	0.187	60.886	57.204	7.363	1	3.33E-03	150
18	0.0519	0.0247	0.1142	0.0201	0.1661	0.0212	0.3127	0.1277	0.5016	0.205	61.803	57.204	9.196	1	1.21E-03	150
19	0.0118	0.0125	0.1516	0.0207	0.1634	0.0190	0.0722	0.0756	0.1157	0.121	58.157	57.204	1.906	1	8.37E-02	150
20	0.0235	0.0182	0.1410	0.0217	0.1645	0.0194	0.1430	0.1064	0.2294	0.171	58.593	57.204	2.778	1	4.78E-02	150
21	0.0860	0.0330	0.0953	0.0199	0.1813	0.0257	0.4743	0.1344	0.7608	0.216	61.468	57.204	8.527	1	1.75E-03	150
22	0.0241	0.0196	0.1417	0.0223	0.1658	0.0197	0.1453	0.1135	0.2331	0.182	58.062	57.204	1.716	1	9.51E-02	150
23	0	0.010	0.165	0.022	0.165	0.019	1.00E-06	0.063	2.00E-06	0.100	57.204	57.204	0	1	5.00E-01	150

Chromosome/Region	V(G) Variance	V(G) SE	V(e) Variance	V(e) SE	Vp Variance	Vp SE	V(G)/Vp Variance	V(G)/Vp SE	V(G)/Vp_L Variance	V(G)/Vp_L SE	LogL	LogL0	LRT	df	Pval	n
24	0.040	0.021	0.120	0.019	0.161	0.020	0.251	0.114	0.403	0.183	63.024	57.204	11.64	1	3.23E-04	150
25	0.018	0.016	0.146	0.021	0.164	0.019	0.108	0.096	0.173	0.154	58.304	57.204	2.199	1	6.91E-02	150
26	0.009	0.014	0.156	0.022	0.165	0.019	0.052	0.082	0.083	0.132	57.469	57.204	0.529	1	2.33E-01	150
27	0.064	0.022	0.093	0.017	0.157	0.020	0.407	0.113	0.653	0.181	68.222	57.204	22.035	1	1.34E-06	150
28	0.019	0.020	0.147	0.024	0.166	0.019	0.113	0.118	0.181	0.189	57.48	57.204	0.552	1	2.29E-01	150
29	0.013	0.015	0.151	0.022	0.164	0.019	0.079	0.092	0.127	0.147	57.74	57.204	1.072	1	1.50E-01	150
30	0.011	0.015	0.154	0.022	0.165	0.019	0.066	0.088	0.106	0.141	57.528	57.204	0.648	1	2.10E-01	150
31	0.015	0.015	0.149	0.022	0.164	0.019	0.090	0.091	0.144	0.146	58.026	57.204	1.643	1	9.99E-02	150
32	0.014	0.015	0.150	0.022	0.164	0.019	0.083	0.092	0.134	0.148	57.767	57.204	1.126	1	1.44E-01	150
33	0.009	0.016	0.156	0.024	0.165	0.019	0.052	0.098	0.084	0.157	57.324	57.204	0.239	1	3.12E-01	150
34	0	0.012	0.163	0.021	0.163	0.019	1.00E-06	0.074	2.00E-06	0.119	57.197	57.204	0	1	5.00E-01	150
35	0.007	0.012	0.158	0.021	0.164	0.019	0.040	0.070	0.064	0.113	57.489	57.204	0.569	1	2.25E-01	150
36	0.015	0.015	0.148	0.021	0.163	0.019	0.091	0.088	0.146	0.141	58.373	57.204	2.337	1	6.32E-02	150
37	0.029	0.021	0.136	0.022	0.165	0.020	0.174	0.119	0.278	0.191	58.552	57.204	2.695	1	5.03E-02	150
38	0.021	0.017	0.142	0.021	0.163	0.019	0.129	0.100	0.207	0.160	58.902	57.204	3.395	1	3.27E-02	150
All chromosomes	0.161	0.042	0.000	0.033	0.161	0.020	1.000	0.205	1.604	0.329	68.16	57.204	21.911	1	1.43E-06	150
Chr 18, 37-56Mb	0.069	0.027	0.099	0.017	0.168	0.023	0.409	0.121	0.656	0.194	66.625	57.204	18.842	1	7.10E-06	150
Chr 18, 38-42Mb	0.035	0.020	0.128	0.016	0.164	0.023	0.216	0.102	0.347	0.164	65.627	57.204	16.845	1	2.03E-05	150
Chr 27, 1-9Mb	0.039	0.017	0.112	0.016	0.151	0.019	0.261	0.096	0.419	0.154	69.838	57.204	25.268	1	2.50E-07	150
Chr 27, 5-6Mb	0.025	0.014	0.130	0.016	0.154	0.020	0.160	0.082	0.257	0.131	67.45	57.204	20.492	1	2.99E-06	150
Chr 27, 5-9Mb	0.033	0.016	0.122	0.016	0.154	0.020	0.210	0.088	0.338	0.141	67.666	57.204	20.923	1	2.39E-06	150

Supplementary Data S2 (continued)

(B) Prevalence 0.05

Chromosome/Region	V(G) Variance	V(G) SE	V(e) Variance	V(e) SE	Vp Variance	Vp SE	V(G)/Vp Variance	V(G)/Vp SE	V(G)/Vp_L Variance	V(G)/Vp_L SE	LogL	LogL0	LRT	df	Pval	n
1	0.014	0.019	0.151	0.025	0.165	0.019	0.083	0.116	0.107	0.150	57.425	57.204	0.441	1	2.53E-01	150
2	0.023	0.021	0.142	0.024	0.165	0.019	0.138	0.122	0.179	0.158	58.08	57.204	1.751	1	9.29E-02	150
3	0.049	0.026	0.119	0.022	0.168	0.021	0.292	0.138	0.378	0.178	59.781	57.204	5.153	1	1.16E-02	150
4	0.010	0.016	0.154	0.023	0.164	0.019	0.062	0.095	0.081	0.123	57.451	57.204	0.494	1	2.41E-01	150
5	0.049	0.025	0.115	0.022	0.165	0.020	0.300	0.136	0.388	0.176	60.232	57.204	6.056	1	6.93E-03	150
6	0.014	0.017	0.151	0.023	0.165	0.019	0.083	0.099	0.108	0.128	57.668	57.204	0.927	1	1.68E-01	150
7	0.037	0.024	0.129	0.023	0.165	0.020	0.222	0.133	0.287	0.172	58.88	57.204	3.35	1	3.36E-02	150
8	0.008	0.014	0.156	0.022	0.164	0.019	0.051	0.082	0.065	0.106	57.504	57.204	0.6	1	2.19E-01	150
9	0.000	0.012	0.165	0.023	0.165	0.019	0.002	0.073	0.003	0.095	57.205	57.204	0.001	1	4.88E-01	150
10	0.009	0.013	0.155	0.022	0.164	0.019	0.054	0.081	0.070	0.105	57.592	57.204	0.776	1	1.89E-01	150
11	0.022	0.019	0.142	0.023	0.164	0.019	0.134	0.111	0.173	0.144	58.179	57.204	1.949	1	8.13E-02	150
12	0.040	0.022	0.124	0.021	0.165	0.020	0.245	0.120	0.317	0.155	60.42	57.204	6.431	1	5.61E-03	150
13	0.015	0.016	0.149	0.022	0.164	0.019	0.092	0.095	0.119	0.123	57.911	57.204	1.413	1	1.17E-01	150
14	0.045	0.023	0.119	0.020	0.164	0.021	0.275	0.122	0.356	0.157	61.495	57.204	8.582	1	1.70E-03	150
15	0.007	0.012	0.158	0.022	0.165	0.019	0.040	0.075	0.052	0.097	57.4	57.204	0.392	1	2.66E-01	150
16	0.000	0.011	0.165	0.022	0.165	0.019	0.003	0.068	0.003	0.088	57.205	57.204	0.001	1	4.86E-01	150
17	0.040	0.021	0.123	0.020	0.163	0.020	0.245	0.117	0.317	0.151	60.886	57.204	7.363	1	3.33E-03	150
18	0.052	0.025	0.114	0.020	0.166	0.021	0.313	0.128	0.405	0.165	61.803	57.204	9.196	1	1.21E-03	150
19	0.012	0.013	0.152	0.021	0.163	0.019	0.072	0.076	0.093	0.098	58.157	57.204	1.906	1	8.37E-02	150
20	0.024	0.018	0.141	0.022	0.164	0.019	0.143	0.106	0.185	0.138	58.593	57.204	2.778	1	4.78E-02	150
21	0.086	0.033	0.095	0.020	0.181	0.026	0.474	0.134	0.614	0.174	61.468	57.204	8.527	1	1.75E-03	150
22	0.024	0.020	0.142	0.022	0.166	0.020	0.145	0.114	0.188	0.147	58.062	57.204	1.716	1	9.51E-02	150
23	0	0.010	0.165	0.022	0.165	0.019	1.00E-06	0.063	1.00E-06	0.081	57.204	57.204	0	1	5.00E-01	150
24	0.040	0.021	0.120	0.019	0.161	0.020	0.251	0.114	0.325	0.147	63.024	57.204	11.64	1	3.23E-04	150

Chromosome/Region	V(G) Variance	V(G) SE	V(e) Variance	V(e) SE	Vp Variance	Vp SE	V(G)/Vp Variance	V(G)/Vp SE	V(G)/Vp_L Variance	V(G)/Vp_L SE	LogL	LogL0	LRT	df	Pval	n
25	0.018	0.016	0.146	0.021	0.164	0.019	0.108	0.096	0.140	0.124	58.304	57.204	2.199	1	6.91E-02	150
26	0.009	0.014	0.156	0.022	0.165	0.019	0.052	0.082	0.067	0.106	57.469	57.204	0.529	1	2.33E-01	150
27	0.064	0.022	0.093	0.017	0.157	0.020	0.407	0.113	0.527	0.146	68.222	57.204	22.035	1	1.34E-06	150
28	0.019	0.020	0.147	0.024	0.166	0.019	0.113	0.118	0.146	0.152	57.48	57.204	0.552	1	2.29E-01	150
29	0.013	0.015	0.151	0.022	0.164	0.019	0.079	0.092	0.103	0.119	57.74	57.204	1.072	1	1.50E-01	150
30	0.011	0.015	0.154	0.022	0.165	0.019	0.066	0.088	0.085	0.114	57.528	57.204	0.648	1	2.10E-01	150
31	0.015	0.015	0.149	0.022	0.164	0.019	0.090	0.091	0.116	0.118	58.026	57.204	1.643	1	9.99E-02	150
32	0.014	0.015	0.150	0.022	0.164	0.019	0.083	0.092	0.108	0.119	57.767	57.204	1.126	1	1.44E-01	150
33	0.009	0.016	0.156	0.024	0.165	0.019	0.052	0.098	0.068	0.127	57.324	57.204	0.239	1	3.12E-01	150
34	0	0.012	0.163	0.021	0.163	0.019	1.00E-06	0.074	1.00E-06	0.096	57.197	57.204	0	1	5.00E-01	150
35	0.007	0.012	0.158	0.021	0.164	0.019	0.040	0.070	0.051	0.091	57.489	57.204	0.569	1	2.25E-01	150
36	0.015	0.015	0.148	0.021	0.163	0.019	0.091	0.088	0.118	0.113	58.373	57.204	2.337	1	6.32E-02	150
37	0.029	0.021	0.136	0.022	0.165	0.020	0.174	0.119	0.225	0.154	58.552	57.204	2.695	1	5.03E-02	150
38	0.021	0.017	0.142	0.021	0.163	0.019	0.129	0.100	0.167	0.129	58.902	57.204	3.395	1	3.27E-02	150
All	0.161	0.042	0	0.033	0.161	0.020	1.000	0.205	1.294	0.266	68.16	57.204	21.911	1	1.43E-06	150
Chr 18, 37-56Mb	0.069	0.027	0.099	0.017	0.168	0.023	0.409	0.121	0.529	0.157	66.625	57.204	18.842	1	7.10E-06	150
Chr 18, 38-42Mb	0.035	0.020	0.128	0.016	0.164	0.023	0.216	0.102	0.280	0.132	65.627	57.204	16.845	1	2.03E-05	150
Chr 27, 1-9Mb	0.039	0.017	0.112	0.016	0.151	0.019	0.261	0.096	0.338	0.124	69.838	57.204	25.268	1	2.50E-07	150
Chr 27, 5-6Mb	0.025	0.014	0.130	0.016	0.154	0.020	0.160	0.082	0.207	0.106	67.45	57.204	20.492	1	2.99E-06	150
Chr 27, 5-9Mb	0.033	0.016	0.122	0.016	0.154	0.020	0.210	0.088	0.272	0.114	67.666	57.204	20.923	1	2.39E-06	150

Supplementary Data S2 (continued)

(C) Prevalence 0.025

Chromosome/Region	V(G) Variance	V(G) SE	V(e) Variance	V(e) SE	Vp Variance	Vp SE	V(G)/Vp Variance	V(G)/Vp SE	V(G)/Vp_L Variance	V(G)/Vp_L SE	LogL	LogL0	LRT	df	Pval	n
1	0.014	0.019	0.151	0.025	0.165	0.019	0.083	0.116	0.088	0.123	57.425	57.204	0.441	1	2.53E-01	150
2	0.023	0.021	0.142	0.024	0.165	0.019	0.138	0.122	0.147	0.129	58.08	57.204	1.751	1	9.29E-02	150
3	0.049	0.026	0.119	0.022	0.168	0.021	0.292	0.138	0.310	0.146	59.781	57.204	5.153	1	1.16E-02	150
4	0.010	0.016	0.154	0.023	0.164	0.019	0.062	0.095	0.066	0.101	57.451	57.204	0.494	1	2.41E-01	150
5	0.049	0.025	0.115	0.022	0.165	0.020	0.300	0.136	0.318	0.145	60.232	57.204	6.056	1	6.93E-03	150
6	0.014	0.017	0.151	0.023	0.165	0.019	0.083	0.099	0.088	0.105	57.668	57.204	0.927	1	1.68E-01	150
7	0.037	0.024	0.129	0.023	0.165	0.020	0.222	0.133	0.235	0.141	58.88	57.204	3.35	1	3.36E-02	150
8	0.008	0.014	0.156	0.022	0.164	0.019	0.051	0.082	0.054	0.087	57.504	57.204	0.6	1	2.19E-01	150
9	0.000	0.012	0.165	0.023	0.165	0.019	0.002	0.073	0.002	0.078	57.205	57.204	0.001	1	4.88E-01	150
10	0.009	0.013	0.155	0.022	0.164	0.019	0.054	0.081	0.057	0.086	57.592	57.204	0.776	1	1.89E-01	150
11	0.022	0.019	0.142	0.023	0.164	0.019	0.134	0.111	0.142	0.118	58.179	57.204	1.949	1	8.13E-02	150
12	0.040	0.022	0.124	0.021	0.165	0.020	0.245	0.120	0.260	0.127	60.42	57.204	6.431	1	5.61E-03	150
13	0.015	0.016	0.149	0.022	0.164	0.019	0.092	0.095	0.098	0.101	57.911	57.204	1.413	1	1.17E-01	150
14	0.045	0.023	0.119	0.020	0.164	0.021	0.275	0.122	0.292	0.129	61.495	57.204	8.582	1	1.70E-03	150
15	0.007	0.012	0.158	0.022	0.165	0.019	0.040	0.075	0.043	0.079	57.4	57.204	0.392	1	2.66E-01	150
16	0.000	0.011	0.165	0.022	0.165	0.019	0.003	0.068	0.003	0.072	57.205	57.204	0.001	1	4.86E-01	150
17	0.040	0.021	0.123	0.020	0.163	0.020	0.245	0.117	0.260	0.124	60.886	57.204	7.363	1	3.33E-03	150
18	0.052	0.025	0.114	0.020	0.166	0.021	0.313	0.128	0.332	0.135	61.803	57.204	9.196	1	1.21E-03	150
19	0.012	0.013	0.152	0.021	0.163	0.019	0.072	0.076	0.077	0.080	58.157	57.204	1.906	1	8.37E-02	150
20	0.024	0.018	0.141	0.022	0.164	0.019	0.143	0.106	0.152	0.113	58.593	57.204	2.778	1	4.78E-02	150
21	0.086	0.033	0.095	0.020	0.181	0.026	0.474	0.134	0.503	0.143	61.468	57.204	8.527	1	1.75E-03	150
22	0.024	0.020	0.142	0.022	0.166	0.020	0.145	0.114	0.154	0.120	58.062	57.204	1.716	1	9.51E-02	150
23	0	0.010	0.165	0.022	0.165	0.019	1.00E-06	0.063	1.00E-06	0.066426	57.204	57.204	0	1	5.00E-01	150

Chromosome/Region	V(G) Variance	V(G) SE	V(e) Variance	V(e) SE	Vp Variance	Vp SE	V(G)/Vp Variance	V(G)/Vp SE	V(G)/Vp_L Variance	V(G)/Vp_L SE	LogL	LogL0	LRT	df	Pval	n
24	0.040	0.021	0.120	0.019	0.161	0.020	0.251	0.114	0.266	0.121	63.024	57.204	11.64	1	3.23E-04	150
25	0.018	0.016	0.146	0.021	0.164	0.019	0.108	0.096	0.115	0.102	58.304	57.204	2.199	1	6.91E-02	150
26	0.009	0.014	0.156	0.022	0.165	0.019	0.052	0.082	0.055	0.087	57.469	57.204	0.529	1	2.33E-01	150
27	0.064	0.022	0.093	0.017	0.157	0.020	0.407	0.113	0.432	0.119	68.222	57.204	22.035	1	1.34E-06	150
28	0.019	0.020	0.147	0.024	0.166	0.019	0.113	0.118	0.120	0.125	57.48	57.204	0.552	1	2.29E-01	150
29	0.013	0.015	0.151	0.022	0.164	0.019	0.079	0.092	0.084	0.097	57.74	57.204	1.072	1	1.50E-01	150
30	0.011	0.015	0.154	0.022	0.165	0.019	0.066	0.088	0.070	0.093	57.528	57.204	0.648	1	2.10E-01	150
31	0.015	0.015	0.149	0.022	0.164	0.019	0.090	0.091	0.095	0.097	58.026	57.204	1.643	1	9.99E-02	150
32	0.014	0.015	0.150	0.022	0.164	0.019	0.083	0.092	0.088	0.098	57.767	57.204	1.126	1	1.44E-01	150
33	0.009	0.016	0.156	0.024	0.165	0.019	0.052	0.098	0.056	0.104	57.324	57.204	0.239	1	3.12E-01	150
34	0	0.012	0.163	0.021	0.163	0.019	1.00E-06	0.074	1.00E-06	0.079	57.197	57.204	0	1	5.00E-01	150
35	0.007	0.012	0.158	0.021	0.164	0.019	0.040	0.070	0.042	0.074	57.489	57.204	0.569	1	2.25E-01	150
36	0.015	0.015	0.148	0.021	0.163	0.019	0.091	0.088	0.097	0.093	58.373	57.204	2.337	1	6.32E-02	150
37	0.029	0.021	0.136	0.022	0.165	0.020	0.174	0.119	0.184	0.126	58.552	57.204	2.695	1	5.03E-02	150
38	0.021	0.017	0.142	0.021	0.163	0.019	0.129	0.100	0.137	0.106	58.902	57.204	3.395	1	3.27E-02	150
All	0.161	0.042	0	0.033	0.161	0.020	1.000	0.205	1.061	0.218	68.16	57.204	21.911	1	1.43E-06	150
Chr 18, 37-56Mb	0.069	0.027	0.099	0.017	0.168	0.023	0.409	0.121	0.434	0.128	66.625	57.204	18.842	1	7.10E-06	150
Chr 18, 38-42Mb	0.035	0.020	0.128	0.016	0.164	0.023	0.216	0.102	0.229	0.108	65.627	57.204	16.845	1	2.03E-05	150
Chr 27, 1-9Mb	0.039	0.017	0.112	0.016	0.151	0.019	0.261	0.096	0.277	0.102	69.838	57.204	25.268	1	2.50E-07	150
Chr 27, 5-6Mb	0.025	0.014	0.130	0.016	0.154	0.020	0.160	0.082	0.170	0.087	67.45	57.204	20.492	1	2.99E-06	150
Chr 27, 5-9Mb	0.033	0.016	0.122	0.016	0.154	0.020	0.210	0.088	0.223	0.093	67.666	57.204	20.923	1	2.39E-06	150
Chr 27, 8-9Mb	0.025	0.015	0.134	0.016	0.159	0.021	0.156	0.084	0.166	0.089	65.208	57.204	16.008	1	3.15E-05	150

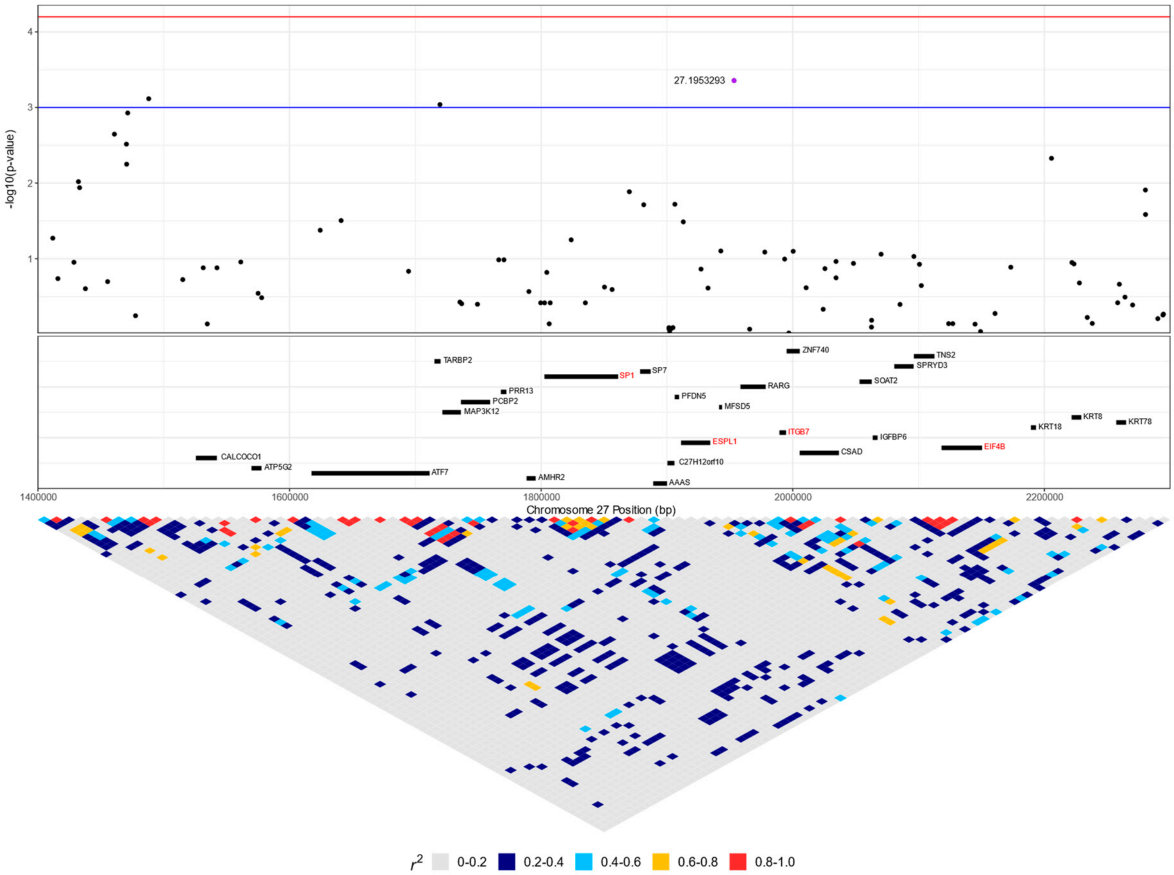

Supplementary Figure S1. Regional association plot for a region of interest in chromosome 27 (1.4 to 2.3 Mb). The top of the plot indicates -$\log_{10}$ p-values for each SNP from the mixed linear model association analysis from the quantitative phenotype GWAS. Red and blue lines indicate q-value cut-offs of 0.05 and 0.1, respectively. The top associated SNP in this region is highlighted (purple dot). The protein-coding genes (CanFam3.1, assembly GCF_000002285.3) in the region, fetched from NCBI, are shown in the middle box, with viral-infection or cancer-associated genes identified through KEGG pathways highlighted in red. Linkage disequilibrium (r^2) in the region is shown in the bottom triangle.

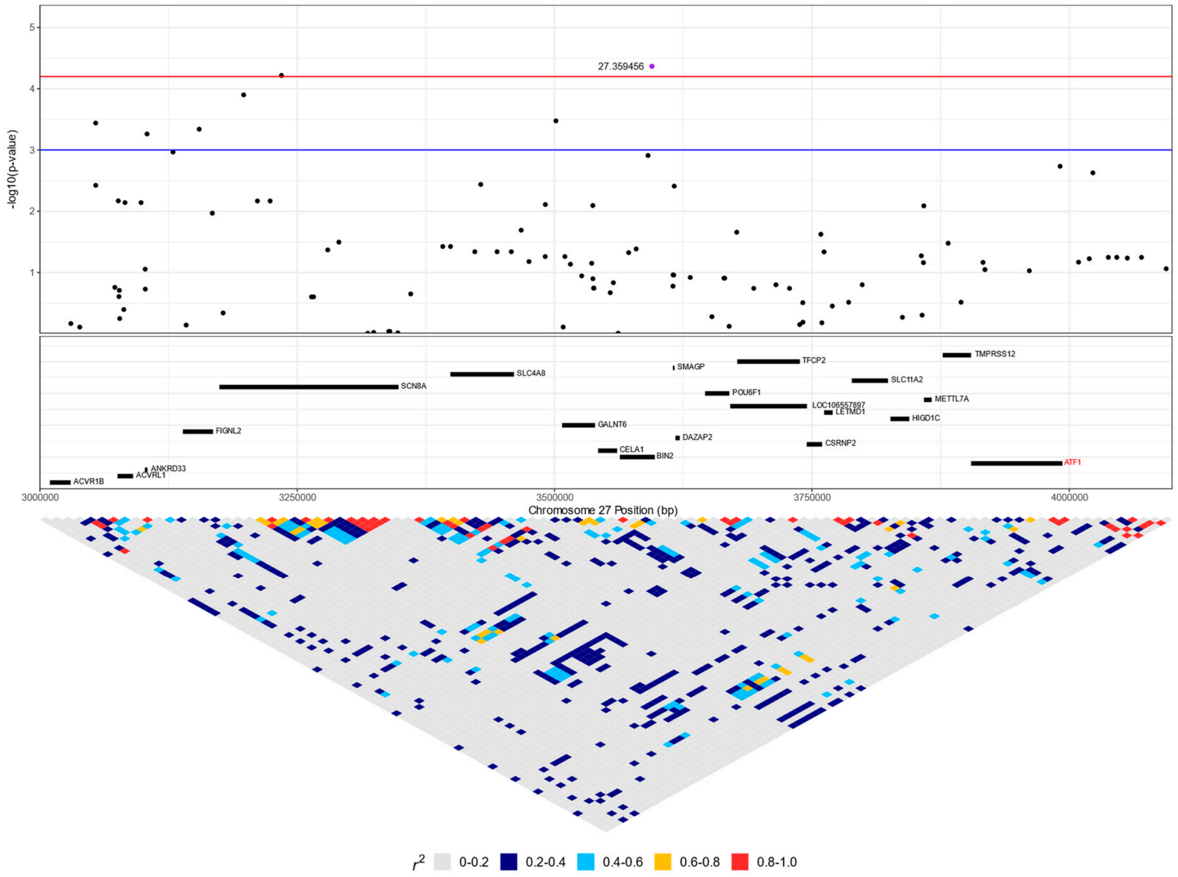

Supplementary Figure S2. Regional association plot for the region around the second most significant SNP in chromosome 27 (3 to 4.1 Mb). The top of the plot indicates -$\log_{10}$ p-values for each SNP from the mixed linear model association analysis from the quantitative phenotype GWAS. Red and blue lines indicate q-value cut-offs of 0.05 and 0.1, respectively. The top associated SNP in this region is highlighted (purple dot). The protein-coding genes (CanFam3.1, assembly GCF_000002285.3) in the region, fetched from NCBI, are shown in the middle box, with viral-infection or cancer-associated genes identified through KEGG pathways highlighted in red. Linkage disequilibrium (r^2) in the region is shown in the bottom triangle.

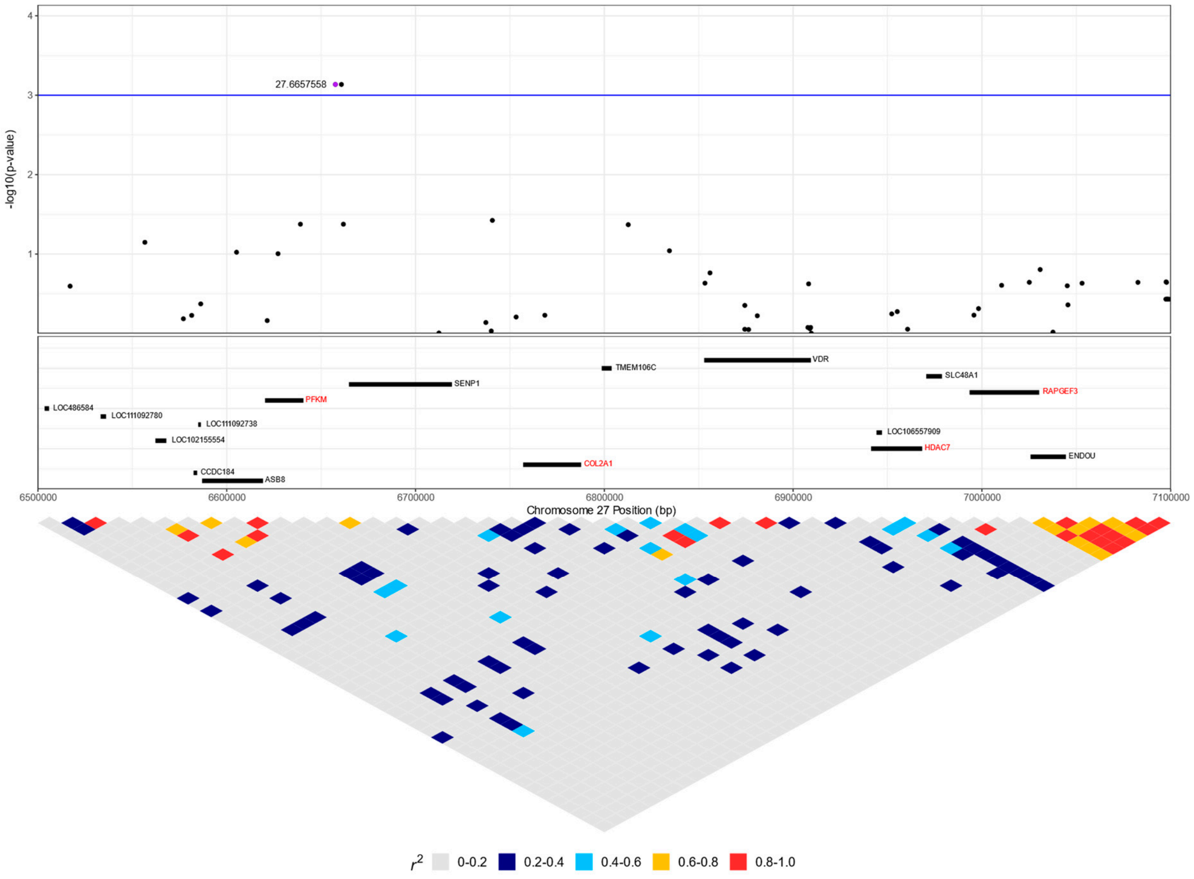

Supplementary Figure S3. Regional association plot for a region of interest in chromosome 27 (6 to 7.1 Mb).The top of the plot indicates -$\log_{10}$ p-values for each SNP from the mixed linear model association analysis from the quantitative phenotype GWAS. The blue line indicates a q-value cut-off of 0.1. The top associated SNP in this region is highlighted (purple dot). The protein-coding genes (CanFam3.1, assembly GCF_000002285.3) in the region, fetched from NCBI, are shown in the middle box, with viral-infection or cancer-associated genes identified through KEGG pathways highlighted in red. Linkage disequilibrium (r^2) in the region is shown in the bottom triangle.

Supplementary Table S1. Linear discriminant analysis output from a backwards stepwise regression between cases and controls for the top 100 SNPs in the mixed linear model analysis using a quantitative phenotype. A model with the top 100 SNPs is also included for comparison. A total of 54 SNPs (bold) were in common with those retained from the stepwise regression of the top 100 SNPs from the binary phenotype GWAS. DF = degrees of freedom.

Model	Top 100 SNPs	Backwards step regression
No. of SNPs	100	74
Residual standard error	0.1615 on 6 DF	0.1013 on 16 DF
Multiple R2	0.9936	0.9933
Adjusted R2	0.8421	0.9379
F-statistic	6.556 on 143 and 6 DF	17.91 on 133 and 16 DF
p-value	0.0117	3.79E-08
SNPs	2:80997329<br>2:81324749<br>2:81332407	**2:80997329**<br>**2:81324749**
	3:7977474<br>3:67414463<br>3:71632869<br>3:71640698<br>3:71691367	3:7977474<br>3:67414463<br>**3:71632869**<br><br>3:71691367
	5:9379432<br>5:9499753	5:9379432<br>**5:9499753**
	7:63218541<br>7:67274886<br>7:67526042	7:63218541<br>**7:67274886**<br>7:67526042
	9:34803797<br>9:35274622<br>9:49959123	**9:34803797**<br>9:35274622<br>9:49959123
	12:27717563<br>12:35491860<br>12:35507522<br>12:35527747<br>12:69769664	12:27717563<br><br>**12:35507522**<br>**12:35527747**<br>12:69769664
	13:17399337<br>13:18910196<br>13:18913685<br>13:20734477	**13:17399337**<br>**13:18910196**
	14:3672303<br>14:3673803<br>14:3693671	14:3672303

Model	Top 100 SNPs	Backwards step regression
	14:3699542	
	14:3716974	
	14:3738150	
	14:3945297	**14:3945297**
	14:4095927	
	14:53561013	**14:53561013**
	14:54260890	**14:54260890**
	15:47616623	**15:47616623**
	18:37249960	18:37249960
	18:37737740	**18:37737740**
	18:37862012	**18:37862012**
	18:37867871	
	18:38233567	**18:38233567**
	18:38326192	18:38326192
	18:38350947	**18:38350947**
	18:38456518	
	18:38502268	**18:38502268**
	18:38507461	
	18:38510335	
	18:38704682	**18:38704682**
	18:38719709	**18:38719709**
	18:39140112	**18:39140112**
	18:39177075	18:39177075
	18:40281122	**18:40281122**
	18:40286669	**18:40286669**
	18:40418483	**18:40418483**
	18:40653765	**18:40653765**
	18:40667579	
	18:41229735	**18:41229735**
	18:41436427	**18:41436427**
	18:41854962	**18:41854962**
	18:55380537	18:55380537
	21:37837424	**21:37837424**
	21:37847374	
	21:39045733	
	21:39068050	
	21:39080288	
	22:15750444	22:15750444
	22:39139988	**22:39139988**
	22:39523927	**22:39523927**

Model	Top 100 SNPs	Backwards step regression
	22:40039487	**22:40039487**
	24:40229170	**24:40229170**
	25:15529304	**25:15529304**
	27:1953293	27:1953293
	27:3054141	27:3054141
	27:3154712	**27:3154712**
	27:3197949	**27:3197949**
	27:3234647	**27:3234647**
	27:3501246	**27:3501246**
	27:3594560	27:3594560
	27:5112245	**27:5112245**
	27:5364442	**27:5364442**
	27:5467028	**27:5467028**
	27:5603116	**27:5603116**
	27:8331252	**27:8331252**
	27:8646723	**27:8646723**
	27:8767784	**27:8767784**
	27:8842830	**27:8842830**
	27:8883501	**27:8883501**
	27:8884575	
	27:8892980	
	28:40578166	28:40578166
	28:40672357	**28:40672357**
	28:40681845	**28:40681845**
	28:41018541	
	28:41020381	
	31:14627591	**31:14627591**
	33:22443454	33:22443454
	33:23263219	
	36:13452474	**36:13452474**
	36:13453583	
	36:16411362	**36:16411362**

Supplementary Table S2. Linear discriminant analysis output from a backwards stepwise regression for the top 100 SNPs in the mixed linear model analysis using a binary phenotype. A model with the top 100 SNPs is also included for comparison. A total of 54 SNPs (bold) were in common with those retained from the stepwise regression of the top 100 SNPs from the quantitative phenotype GWAS. DF = degrees of freedom.

**Model**	**Top 100 SNPs**	**Backwards step regression**
No. of SNPs	100	79
Residual standard error	0.1416 on 9 DF	0.1186 on 13 DF
Multiple R2	0.9927	0.9926
Adjusted R2	0.8786	0.9147
F-statistic	8.701 on 140 and 9 DF	12.75 on 136 and 13 DF
p-value	7.03E-04	5.35E-06
SNPs	2:80997329<br>2:81324749<br>2:81332407	**2:80997329**<br>**2:81324749**
	3:71632869<br>3:71640698<br>3:71691367<br>3:77341029	**3:71632869**<br><br>**3:71691367**<br>3:77341029
	5:9499753<br>5:14836517	**5:9499753**<br>5:14836517
	7:67274886	**7:67274886**
	9:34803797	**9:34803797**
	10:1868661<br>10:2493438	10:1868661
	12:33397646<br>12:35491860<br>12:35507522<br>12:35527747	12:33397646<br>12:35491860<br>**12:35507522**<br>**12:35527747**
	13:17399337<br>13:18910196<br>13:18913685<br>13:20734477<br>13:20995480<br>13:20998820	**13:17399337**<br>**13:18910196**<br><br>13:20734477<br>13:20995480
	14:628308<br>14:779271<br>14:3945297<br>14:4095927<br>14:53561013<br>14:54260890	14:628308<br>14:779271<br>**14:3945297**<br><br>**14:53561013**<br>**14:54260890**
	15:47616623	**15:47616623**

	17:51052810	17:51052810
	18:37737740	**18:37737740**
	18:37862012	**18:37862012**
	18:37867871	
	18:38233567	**18:38233567**
	18:38350947	**18:38350947**
	18:38456518	
	18:38502268	**18:38502268**
	18:38507461	
	18:38510335	
	18:38704682	**18:38704682**
	18:38719709	**18:38719709**
	18:39140112	**18:39140112**
	18:39727438	18:39727438
	18:40281122	**18:40281122**
	18:40286669	**18:40286669**
	18:40418483	**18:40418483**
	18:40653765	**18:40653765**
	18:40667579	
	18:41229735	**18:41229735**
	18:41436427	**18:41436427**
	18:41713196	18:41713196
	18:41726488	18:41726488
	18:41854962	**18:41854962**
	18:42270324	18:42270324
	21:37837424	**21:37837424**
	21:37847374	
	21:39045733	21:39045733
	21:39068050	
	21:39080288	
	21:39399551	
	21:39409009	
	22:39139988	**22:39139988**
	22:39523927	**22:39523927**
	22:40039487	**22:40039487**
	24:34542285	24:34542285
	24:40229170	**24:40229170**
	25:15529304	**25:15529304**
	26:36337542	26:36337542
	27:3154712	**27:3154712**
	27:3197949	**27:3197949**
	27:3234647	**27:3234647**

	27:3501246	**27:3501246**
	27:3594560	
	27:5112245	**27:5112245**
	27:5364442	**27:5364442**
	27:5467028	**27:5467028**
	27:5478927	27:5478927
	27:5512765	27:5512765
	27:5603116	**27:5603116**
	27:5806033	27:5806033
	27:5817551	
	27:5822515	
	27:7188905	27:7188905
	27:8331252	**27:8331252**
	27:8646723	**27:8646723**
	27:8767784	**27:8767784**
	27:8842830	**27:8842830**
	27:8883501	**27:8883501**
	27:8884575	
	27:8892980	27:8892980
	27:11672173	27:11672173
	28:28639313	28:28639313
	28:40672357	**28:40672357**
	28:40681845	**28:40681845**
	28:40998474	28:40998474
	31:14627591	**31:14627591**
	36:13452474	**36:13452474**
	36:13453583	
	36:16411362	**36:16411362**
